# Supplementary material for: Prognostic factors influencing HIV-free survival among infants enrolled for HIV early infant diagnosis services in selected hospitals in Nairobi County, Kenya
Source: PLoS One. 2023 Oct 4;18(10):e0292427. doi: 10.1371/journal.pone.0292427 (PMC10550113; doi:10.1371/journal.pone.0292427)
Supplement: S1 Questionnaires — (PDF) [file pone.0292427.s001.pdf]

Appendix II: Mother-infant pair recruitment questionnaire

A: Semi-Structured Questionnaire for HIV positive women at recruitment

(A) Basic information

Questionnaire Serial Number			
Research assistant name			
Date of interview	Day	Month	Year
Mothers CCC number			
Infant HEI number<br>Infant MBP number			

(B) Socio-Demographic information

1. Where is your place of residence?
   a) Sub-County…………………………Location……………Estate …………….
2. Mobile number of mother…………………………….
3. Mobile number of next of kin and relationship……………………
4. Age in completed years

   a) 18-24 [ ]
   b) 25-34 [ ]
   c) 35-44 [ ]

5. What is the highest level of education completed?
   a) ≤Primary education [ ]
   b) ≥Secondary education [ ]
6. What is the size of your family or the number of people who sleep in your house?
   a) 2-5 [ ]
   b) 6-8 [ ]

7. What is your current marital status?
    a) Single [ ]
    b) Married [ ]
8. What is your current employment status?
    a) Formal employment [ ]
    b) In-formal employment [ ]
    c) Unemployed [ ]
9. What is your monthly household income in Ksh.?
    a) ≤6000 [ ]
    b) 6001-12000 [ ]
    c) ≥ 12001-18000 [ ]

B. Maternal biomedical information at baseline (Confirm from CCC file/maternal ANC booklet)

INDICATOR	STATUS	DATE TAKEN
**Confirm from mothers CCC file**		
Date when the mother was 1st initiated on ART		
Weight (Kgs)		
Height (Centimetres)		
BMI (Kg/M^2)		
CD4 Count (Cells/mm3)		
Viral Load ((IU/mL)		
HB Level (g/dl)		
HIV staging		
Mother's adherence to the last clinic given	Appointment date:<br>Visit date:	
Presence of opportunistic infections		
Presence of co-infections		
**Confirm from mothers ANC booklet**		
Mode of delivery for this infant		
Gestation at delivery for this infant		
Gestation of pregnancy at **1st** PMTCT/ANC visit for this infant		
Number of PMTCT visits during pregnancy for this infant		
Number of appointments missed during pregnancy		

C. Information on HIV status and disclosure status ***(please remember that whatever will be discussed here will be confidential)***

10. Which year were you confirmed to be HIV positive
    a. > 2years prior to study onset [ ]
    b. ≤ 2years prior to study onset [ ]
11. Where were you tested for HIV?
    a) At the hospital during antenatal care (this pregnancy) [ ]
    b) At hospital when sick [ ]
    c) At VCT facility [ ]
    d) Provider Initiated Testing and Counseling (PITC) [ ]
    e) Other (Specify) ………………………………………
12. Do you have a partner? (confirm response with question 7)
    a) Yes
    b) No

    If No, skip to question 17, If yes continue with question 13

13. Have you disclosed your HIV status to your partner?
    a) Yes [ ]
    b) No [ ]
14. Do you know your partner's HIV status?
    a) Yes [ ]
    b) No [ ]

    If No, skip to question 17
15. What is your partner’s status?
    a) Positive [ ]
    b) Negative [ ]

16. How did your spouse/partner know his HIV status?

a) Own voluntary testing [ ]
b) After knowledge of the mother's HIV status [ ]
c) Routine medical check-up [ ]
d) Others (Specify)…………………………………………………….

D. Information on maternal history during PMTCT attendance and ART adherence

17. Did you attend any PMTCT visits during this pregnancy?

a) Yes [ ]
b) No [ ]

If yes, how many visits for the entire pregnancy? (Indicate number)…………

If No go to question 19

18. What services were you given during the PMTCT visit (let the mother give you the responses then probe by reading out responses a-f)

a) HIV testing [ ]
b) Pre and post-test counseling [ ]
c) ARVs [ ]
d) Prophylaxis (Cotrimoxazole) [ ]
e) None [ ]
f) Others (Indicate)……………………………………………………

19. Have you been given any ART during this pregnancy?

a) Yes [ ]
b) No [ ]

If Yes go to question 20 if No go to the Morisky Scale

20. What stage in your pregnancy were the ART administered (Gestation at first PMTCT visit)?
    a) 1st Trimester [ ]
    b) 2nd Trimester [ ]
    c) 3rd Trimester [ ]

Morisky Medication ART adherence scale (MMAS-8) - Circle appropriately

	QUESTION	YES	NO
1.	Do you sometimes find it difficult to remember to take your ART daily?	1	0
2.	Do you ever forget to take your medicine sometimes?	1	0
3.	Sometimes if you feel worse when you take the medicine, do you stop taking it?	1	0
4.	When you feel better, do you sometimes stop taking your medicine?	1	0
5.	Did you take your medicine yesterday?	0	1
6.	When you feel like your symptoms are under control, do you sometimes stop taking your medicine?	1	0
7.	Taking medication every day can be a real inconvenience for some people. Do you ever feel under pressure about sticking to your treatment plan?	1	0
8.	How often do you have difficulty remembering to take all your medications? (Please circle the correct number)<br><br>A) Never/Rarely<br>B) Once in a while<br>C) Sometimes<br>D) Usually<br>E) All the time	POINTS:<br><br>A) 0<br>B) ¼<br>C) ½<br>D) ¾<br>E) 1	

Adapted from guidelines on use of ART and Prevention of HIV infection in Kenya – 2018 Edition

21. Have you been hospitalized during this pregnancy?

   a) Yes [ ]

   b) No [ ]

   If yes, indicate reason(s)……………………………………………

   If no, go to question 23, If Yes continue with number 22

22. Indicate the number of times you have been hospitalized during this pregnancy? (Indicate number here)………………………………………………..

E. Maternal knowledge and attitude towards EID

23. Do you have any knowledge on how to reduce the chances of HIV transmission to your infant?

   a) Yes [ ]

   b) No [ ]

   If No go to question 25, If yes continue with question 24

24. You can reduce the chances of HIV transmission to your infant by? (Tick appropriately)

   a) Appropriate feeding practices [ ]

   b) Prophylaxis for the infant immediately after birth [ ]

   c) ART for the mother during pregnancy and thereafter[ ]

   d) Early HIV diagnosis for the mother [ ]

   e) Appropriate mode of hospital deliver [ ]

   f) None of the above [ ]

   g) Other (Indicate) ………………………………………………..

25. Would you be willing to bring your infant for HIV testing using PCR to determine their HIV status at 4-6 weeks, 6 months, and 12 months and any other prescribed time thereafter?

   a) Yes [ ]

   b) No [ ]

   If No, indicate reason(s)……………………………………………

26. Would you be willing to give your child ARVs for PMTCT of HIV for the prescribed duration every day without fail?

a) Yes [ ]

b) No [ ]

If No, indicate reason(s)………………………………………

27. If your baby tests HIV positive by PCR, would you be willing to ensure that your baby takes ART for the rest of his/her life?

a) Yes [ ]

b) No [ ]

If No, Indicate reason(s)………………………………………

F. Maternal knowledge of infant feeding practices

28. Do you know the available feeding options for your infant?

a) Yes [ ]

b) No [ ]

29. Have you been educated on the available feeding options for your infant?

a) Yes [ ]

b) No [ ]

If No go to question 31, if yes continue with question 30

30. What was the source of your information on feeding practices for your infant?

a) ANC Clinics [ ]

b) Other departments in the healthcare facility [ ]

c) Spouse [ ]

d) Extended family members [ ]

e) Friends [ ]

f) Same as how I fed my other infants [ ]

g) Others (Indicate)………………………………………..

31. What feeding option will you settle for your infant in the first 6 months of life?

a) Exclusive breastfeeding for the first 6 months [ ]

b) Mixed feeding for instance breastfeeding and other substitutes such as water, or boiled cow's milk from birth [ ]

c) Formula feeding from birth without giving breast milk [ ]

d) Breastfeeding and formula feeding from birth [ ]

e) Boiled animal milk from birth without breastfeeding [ ]

f) Heat-treated breast milk for first 6 months [ ]

g) Other (Explain)……………………………………………

G: Infant feeding practices

32. What was the <u>first milk</u> that you gave (or someone else gave) to your infant after birth? (Select only one response)

a) Breast milk [ ]

b) Formula milk [ ]

c) Animal milk (goat, cow, camel) [ ]

33. How soon after delivery was this milk given to your baby?

a) Less than one hour [ ]

b) 1-4 hours [ ]

c) 4-12 hours [ ]

d) More than 12 hours [ ]

e) I cannot recall [ ]

34. Is (name) currently breastfeeding?

a) Yes [ ]

b) No [ ]

If yes continue with question 35, If No skip to question 36

35. Is (name) breastfeeding exclusively?

a) Yes [ ]

b) No [ ]

If Yes, continue with question 36, If No skip to question 37

36. What age did you introduce the foods?

a) Immediately after birth [ ]

b) When baby was < 6 months old [ ]

c) Only before my breasts started producing milk [ ]

d) Other (Indicate)……………………………………….

37. What kind of foods are you giving to your baby? Tick all that apply.

a) Plain water [ ]

b) Infant formula milk [ ]

c) Animal milk (Cow's/goat/camel milk) [ ]

d) Thin porridge [ ]

e) Mashed Fruits [ ]

f) Mashed potatoes [ ]

g) Others (Specify)……………………………………………

38. How did you make the above decision? (Select only One response)

a) By yourself- no discussion with anyone [ ]

b) After discussion with someone else [ ]

c) Your family or extended family members told you [ ]

d) Healthcare provider told you [ ]

e) Same as previous child [ ]

f) Other (Indicate)………………………………………………

H: Initiation into care and treatment

39. Briefly describe in your understanding, the services that will be offered to you and your infant as you go through the EID process……………………………..………………………………………………

40. Is there any reason as to why you would not want your infant to be initiated into care and/or treatment with ARVs and cotrimoxazole? ....…………………………………………………

41. At what age was your infant initiated into the EID program? (Confirm response from MCH booklet)

a) At birth [ ]
b) 1 day to 4 weeks [ ]
c) Between 4-6 weeks [ ]
d) >6 weeks but <10 weeks [ ]
e) >10 weeks but <6months [ ]
f) >6months but <12 months [ ]

42. At what age did your infant receive the first PCR results? (If no PCR results **End questionnaire**)

a) At birth [ ]
b) Between 4-6 weeks [ ]
c) >6 weeks but <10 weeks [ ]
d) >10 weeks but <6months [ ]
e) >6months but <12 months [ ]
f) No PCR results received (If no PCR results **End questionnaire**) [ ]

43. What were the results of the PCR test offered to your infant?

a) Positive [ ]
b) Negative [ ]
c) No PCR results given [ ]

44. Is your infant initiated on ART Therapy?

a) Yes [ ]
b) No [ ]

If No End the interview here, If yes continue with question 45

45. At what age was your infant initiated on ART?

g) At birth [ ]
h) Between 4-6 weeks [ ]
i) >6 weeks but <10 weeks [ ]
j) >10 weeks but <6month [ ]
k) >6months but <12 months [ ]

END OF QUESTIONNAIRE. THANK YOU FOR YOUR PARTICIPATION!

Appendix III: Infant Follow-Up 6weeks, 10weeks, 14weeks, 6months, and 12 months.

A: Child characteristics (Confirm from MCH booklet)

(A) Basic information

Questionnaire Serial Number			
Research assistant name			
Date of interview	Day	Month	Year
Mothers CCC number			
Infant HEI number<br><br>Infant MBP number			

**POSITIVE PCR INFANT**			**NEGATIVE PCR INFANT**		
Indicator	Status	Date taken	Indicator	Status	Date taken
Sex of infant			Sex of infant		
Age of infant (weeks)			Age of infant (weeks)		
Birth Weight(Kg)			Birth Weight(Kg)		
Progressive (Kg)			Progressive (Kg)		
Height (cm)			Height (cm)		
Head circumference			Head circumference		
(MUAC) in (cm)			(MUAC) in (cm)		
PCR status			PCR status		
CD4 % (cells/mm^3)			-		
Viral load count (IU/mL)			-		
Co-infections			-		
ART			ART prophylaxis (up to 12 weeks)		
CTX			CTX		
Other medication			Other medication		
Hospitalization(s)			Hospitalization(s)		
Other illnesses			Other illnesses		
Feeding practice			Feeding practice		
PCR result at 6 weeks	Date recieved:		PCR result at 6 weeks	Date received:	

Appendix IV: Data abstraction tool for maternal follow-up at 6 and 12 months

(A) Basic information

Questionnaire Serial Number			
Researchers initials			
Date of interview	Day	Month	Year
Mothers CCC number			
Infant HEI number<br><br>Infant MBP number			

(B) Follow-up of the mother at 6 months and 12 months

Check from mother's CCC file and update

INDICATOR	STATUS	DATE TAKEN
Weight (Kgs)		
Height (centimeters)		
BMI (Kg/M^2)		
CD4 Count ($cells/mm^3$)		
Viral Load (IU/mL)		
HB Level (g/dl)		
HIV staging		
Mother's adherence to the last clinic given		
Presence of opportunistic infections		
Presence of co-infections		
ART mother is taking		

Note: Ensure to administer the Morisky Medication ART Adherence Scale (MMAS-8) to confirm ART adherence.
