# Supplementary material for: Prognostic factors influencing HIV-free survival among infants enrolled for HIV early infant diagnosis services in selected hospitals in Nairobi County, Kenya
Source: PLoS One. 2023 Oct 4;18(10):e0292427. doi: 10.1371/journal.pone.0292427 (PMC10550113; doi:10.1371/journal.pone.0292427)
Supplement: Attachment — Submitted filename: Response to Reviewers..pdf [file pone.0292427.s006.pdf]

<table>
<tr><th colspan="2">Response to reviewers</th></tr>
<tr><th>Reviewers Comments</th><th>Changes effected by the PI</th></tr>
<tr><td>Overall –
• “HIV-positive” and “HIV-infected” are no longer politically correct terminology; suggest using “women living with HIV (WLHA) throughout”. Also suggest using “HIV-exposed infants (HEI)” as they are the subject of a growing field of research – this will make your publication easier to find for those doing literature reviews
• The sub-headings in the paper read like a thesis or a dissertation – specifically, the methods section has many more sub-headings than needed for a manuscript. I suggest looking at similar papers in the literature (especially within this journal), and see what subheadings are consistently used. Style your paper the same way; it’s okay if not everything fits in</td><td>Amended throughout the document.

Unnecessary sub-titles removed more so in chapter 3.</td></tr>
<tr><td>Title –
• Why ‘prognostic’? Suggest removing the word throughout the paper and just say ‘factors’. Your title indicates survival is the end-point, but your methods indicate HIV-positive status as the endpoint. Needs to be clear if it’s one or the other or either.</td><td>Amended i.e. HIV-free survival has been used to describe the endpoint.
The term prognostic has been retained in the title since it means an advance indication of a future event more so whilst undertaking survival analysis. It’s a predictive term which is suitable in this instance.</td></tr>
<tr><td>Abstract –
• Intro - ‘MTCT rate’ needs to be presented with an end time (ie. At 6 weeks of age; 12 months of age; after weaning) – also, technically a rate is measured in person-years (as you’ve done in your results). Suggest using ‘percentage’ or ‘proportion’ in this context
• Methods – Number of facilities? Public and/or private? Sample selected from how many HIV-infected infants (as denominator for proportion chosen). Why one year? Were they all weaned by then, or is the in the</td><td>MTCT rate/period defined as per the WHO guidelines.

- Methods are fully described in the methods section and subsequent sections addressed. There is a word count limit in the abstract hence all the methods used may not be detailed here but have been fully detailed in the methods section.
-Justification for why one year follow up has been</td></tr>
</table>

context of ongoing exposure into the second year of life? What data was collected? Maternal only (as implied in the conclusion of the abstract? Or infant as well, ie. Birthweight, feeding method, weaning age, etc. Ethical approval numbers not needed in the abstract<br>• Results – failure event definition belongs in your methods (but isn't needed in the abstract if space is a challenge). What is "poor infant survival"? Is that death and HIV-infection? Just death? Needs to be very clear. At the moment it reads as though you're using death and HIV-infection interchangeably and conflating the two into 'poor infant survival'<br>• Conclusion – why define recent HIV diagnosis in relation to the 'study onset'. Rather define it as it relates to her own condition and health: ie. 'less than two years before current pregnancy'.	indicated.<br><br>-Ethical approval numbers removed from the abstract.<br>-Failure event maintained in the abstract as this is a very key element to know at the very beginning of the paper.<br>-Failure event clearly defined as infant with a positive PCR test during the study period and not death of infant.<br><br>-Section amended i.e., ≤ 2 years since HIV positive diagnosis.

Introduction	
• Always spell out the first use of acronyms in the main manuscript: HIV, EID, PMTCT – even if spelled out already in the abstract	-Change effected i.e., acronyms spelt out on the first use.
• This is very short and doesn't provide a solid introduction to the topic or the other research in this area. The sentence "there is limited data demonstrating…" in the first paragraph is inaccurate. There's A LOT of research in both MTCT and infant death; you need to use that to your benefit – understand what's out there and what your study brings to the table that is 1. New or additional in the literature, or 2. Confirms what others have found in other countries holds true in Kenya as well	Information in the introduction amended.<br><br>Phrase "there is limited data demonstrating…" removed.<br>More literature has been added to the manuscript.
• This first paragraph is extremely confusing – there's actually more literature on MTCT than on HIV exposed/infected infant survival. This wording doesn't help clarify for the reader immediately which direction you're going in -the implication is HIV infection, but your paper title is "survival" – if your argument later in the paper is to do a survival analysis using HIV infection as your end point, that's different that saying 'survival' and meaning HIV-negative. The 'survival' in 'survival analysis' by definition is the opposite of your end point. But if your end point is HIV-infection, then your 'survival' is 'HIV-free'; you can't use 'survival' in the title or narrative as anything other than being alive; you must use 'HIV-free' or 'HIV-negative', or the reader is left confused	- Component changed to HIV-free survival throughout the document.
• Your references are limited to East Africa research – suggest you broaden your look to southern Africa as well. A lot of PMTCT research (including national level evaluations) have been reported from Malawi, Zimbabwe and South Africa – many of which have	More literature has been included however, the paper also already had referencing from southern Africa among other parts of Africa like Cameroon, Malawi, Guinea, Zimbabwe, Ethiopia, Nigeria, DRC, Rwanda and others like India. More recent data has also been added.

similar analyses as yours. Understanding their findings and how yours do or don't differ will be important to the interpretation of your results section in your discussion<br>• Consider your key findings and think about how the reader needs to be prepared to accept those – ie. Disclosure between partners is really important – there's a paper out of Malawi's national PMTCT evaluation that documents the same, and many other papers showing that male involvement from early in ANC and couples testing is also critical to maternal ART adherence… which of course is what infant infection hinges on – bring these ideas together, along with other key findings.<br>• The paragraph that begins with "Globally, vertical transmission" should be the first paragraph in your paper – it sets the stage for everything else – always start your introduction with the global setting and then narrow down to national and if relevant, sub-national levels. That first sentence also needs to be broken into two sentences..<br>○ Suggestion: This diagram (sourced online) is used widely to describe what the flow of your introduction should look like:	-Although non-disclosure was found to be a factor in this study that influenced HIV-free survival among the infants, it was not considered as a key finding since a lot of interventions have been put in place to facilitate partner disclosure in Kenya. Further research could be done to investigate why disclosure is still a problem. However, as it stands no interventional packages have been put in place for younger mothers less than 18 years and mothers with recent HIV diagnosis. This paper then tries to highlight these special populations to trigger targeted action and reduce MTCT rates in these two populations of mothers seeking PMTCT services.<br><br>-Document flow rearranged to start with the global picture, all the way to the local context i.e. Kenya.
Methods<br>• Sub-headings – should be same size font as the narrative, not bolded, and in italics.<br>• Main headings (ie. Intro, methods) – should be same size font as narrative<br>• If you need a third level heading, make it the same font as the narrative and underline it (no bold or italics)<br>• Female prevalence compared to male prevalence isn't relevant in your methods section – probably doesn't need to be in background either –	I have followed the PLoS guidelines on the website for sub heading and heading font sizes.<br><br>Removed.<br><br>Amended as above.

suggest removing<br>• The details about prevalence in catchment areas could be part of your background section where you talk about Kenya and what's specifically known/unknown about HIV and MTCT in your study area. Don't put site names in your intro; keep those in your methods<br>• "infants born of HIV-positive mothers" should be "HIV-exposed infants"<br>• Sampling – rename subsection to "Study population"<br>• "You need to explain why you chose to use the EID clinic as entry point for the study – why not ANC? Also make sure you include this as a limitation in your discussion section. Your sample excludes infants not identified as HIV-exposed (whether or not their mothers were on ART), and infants who may have died prior to enrolment.<br>• Define CCC<br>• Once the participants were identified from the register, how did you identify them in real life to enroll them? A little more detail is needed here<br>• Exclusion criteria – why exclude those who transferred in within the last year?<br>• Consider putting your inclusion criteria into a single sentence, and your exclusion criteria into a single sentence – think about how to be more concise – ie. "HIV-exposed infants age 2-6 weeks whose mothers were registered in the study facility CCCs and agreed to participate in the study" – three sentences are not needed.<br>• Study variables – you don't need a section listing these out – combine with the data collection tools section and be more clear about what was collected from routine (MOH) data,	There are no site names in the introduction. Site names maintained in the methods.<br><br>Amended to read HIV-exposed infants.<br>Unnecessary sub-titles removed as suggested.<br><br>HIV exposed infants are found in the EID clinics and not ANC clinics as per the Government of Kenya healthcare facility set up. Further, the recruitment was not conducted during pregnancy, rather post-natally i.e., at 2-6 weeks, hence the reason why the infants were recruited in EID clinic.<br><br>CCC defined in the context as Comprehensive Care Clinic.<br>Section on identification added in the text .<br><br>The majority of these mothers did not have their files/records for reference, and it would have been hard to corroborate their information without their records from their previous care center.<br><br>Amended as per suggestion.<br><br>-Independent and dependent variables removed from the narrative.<br>-Information collected by the data collection tools described.

what was collected from your questionnaire, and what labs were collected (or include with routine MOH data if testing was not done separately for the study)<br>• Cohort recruitment and follow-up – move up to come after the sampling section to improve flow<br>• Study validity – you describe what you did and why, but not "how" – did you do any statistical testing for validity? If not, consider removing this section entirely<br>• Data analysis –<br>○ This section needs to describe everything and only what you did – no excess language or definitions of stats techniques. You're missing a description of what you did that aligns with the multiple tables produced in your results:<br>○ Your first paragraph is almost all that's needed: Consider using "conducted" instead of "done" in the second use of "done"; Revise to remove brackets – ie. Remove (mean and median), and reorganize the wording in the K-M sentence ('Kaplan-Meier survival analysis provided…')<br>○ Remove the definition of Cox's regression and all the formulas – these are standard statistical techniques and can be looked up online by anyone who doesn't know them<br>○ 'Modeling' section – this can all be removed. Add AIC as one of your analysis techniques in the first (only) paragraph if wanted.<br>• **Note:** Your methods data analysis description don't match what you show in your results –<br>▪ Why did you separate your multivariable	Amended i.e., moved.<br><br>Study validity described i.e., Cronbach alpha technique used.<br><br>Unnecessary information removed from the data analysis section.<br><br>First paragraph retained.<br><br>Removed<br><br>Section removed.<br><br>Description and table on socio-demographic /economic status included to give more insight of the study population<br>The tables aligned to only have two tables i.e.., socio-demographic factors and PMTCT/disclosure factors

analysis into two separate groupings after the log-rank test? Why not a single model?<br>• And related, why were you limiting yourself to 2 factors in the final model?	All the variables were included in the modelling process and eliminated until the variables that best explained our outcome of interest were identified. This information has been included in the manuscript for clarity. Modelling (both backward and forward) was done using AIC criterion as indicated in the manuscript.
• That should be revised into a one comprehensive model, or alternately, describe what you did and why in this section… you've got this already, but you put these methods in your results section – move the first half of the paragraph that begins "all significant variables at p<0.01…" to your methods section. And then fill in the gaps in my understanding based on the questions immediately above.<br>•	Section has been moved to the methods explaining the modelling process.<br><br>This has been addressed above.<br>Subtitle removed.
○ Why "…to determine the best model using only two prognostic factors"? Why not all variables produced by the model?	All the variables were included in the modelling process and eliminated until the variables that best explained our outcome of interest were identified. This information has been included in the manuscript for clarity. Modelling (both backward and forward) was done using AIC criterion as indicated in the manuscript.
• Study strengths and limitations (remove delimitations) –<br>○ This should be 1-2 paragraphs in your discussion section and not part of your methods	Section amended and moved to methods.
○ 'Wasting' is a chronic condition – not subject to 'rapid fluctuations'. I suggest you stick to just saying 'nutrition parameters'	This parameter has been removed as it was not part of the work presented.
○ If you have a study limitation, you need to think about how it might have affected your results – ie. You recruited at EID entry point so you have almost certainly underestimated HIV	The recruitment for EID for HIV program in Kenya starts from between 2 weeks and 6 weeks post-partum. Hence data of still births is not captured in the EID program and hence not in this study.<br>The limitations identified were also followed by delimitation narratives given as well.

transmission and mortality because you didn't include stillbirths and neonates – this also means you may have overestimated HIV-free survival (and proportion HIV-negative), which would explain why your results are higher than other countries…. These are the kinds of explanations to include in your limitations section.<br>○ A strength you need to include is that you used survival analysis as appropriate for a cohort study, instead of logistic regression as is often done with other cohort studies in this field<br>○ Sample size could influence your results up OR down – you only describe it one way. I think you can reframe this as the small sample size gave you wide confidence intervals (5.5-16.3) which isn't as useful for health policy guidance as narrow CI's.<br>• Ethical considerations – the sentence about women's age should be moved up to your study population section<br>○ Also change your language to be more clear – not 'sought approval', but 'received approval'. There's a big difference. This section really only needs the listing of who provided ethical approvals, and the approval numbers where relevant.	Strength of study included.<br><br>Section amended.<br><br>Section amended.<br><br>Suggestions given were to include all ethical considerations.
Results:<br>• In your results, you ONLY present results – no methods, no opinions, no interpretation<br><br>• This wording: "the study applied right-censoring", and "K-M survival estimate curves were used…", and "K-	Methods and opinions removed. However, interpretations are retained as per the review of other published manuscripts and guidelines.<br><br>Sections moved

M failure estimate curves were used…" , and Mantel log-rank test was used…" all belongs in your methods section	
• You're missing a "Table 1" which provides the basic demographics for mother and infant (age, marital status, education, viral load at enrolment, birthweight, age at weaning, etc)- you need to describe the study population before you jump into the outcomes of the study	Table 1 added which provides the basic demographics for mother and infant
• When you describe a table in your narrative, you need to actually describe the content of the table – not just repeating the table title. Pick the 2-3 most important things from each table and describe them in your results narrative. When you describe, don't repeat all the numbers in the table, but instead writing statements such as "nearly half the infants born to women under age 25 were lost to follow-up, compared to one quarter of infants born to women over age 35" (numbers I made up).	Table narratives amended appropriately<br><br>Amended
• Table 1 (should become 2) –	
○ You need to 'tab' your unbolded row titles so they're indented from the sub-headings within the table	Amended i.e., un bolded
○ Remove "Mantel log-rank tests for" from each sub-heading as the table title says this already	Removed
○ What is "timelines into EID enrolment at 6 weeks"? Is this about whether they were enrolled at a later age? If so, what cut-off did you use to determine late enrolment? 6 weeks? 12 weeks? Suggest revising this so it's clear to the reader	Timeliness into EID has been described as per the GOK, 2016 PMTCT guidelines to be enrollment at or before six weeks of age. Any time after this duration is considered as delayed enrollment.
• Table 2 –	
○ Be careful with your formatting – keep a space between numbers and	Formatting and editing done in all the tables including indenting

brackets for consistency – also indent the values from the variable names such as described above for Table 1(2). I<br>o t's not clear to me why you have two separate Cox regressions – is Table 2 from the enrolment data and Table 3 from 12 mos? If so, the table title is really misleading – I'm guessing from the variables included.	Cox regression tables amended to have one for socio-demographic factors and another for disclosure status and PMTCT factors.
• Table 3 –<br>o By now the reader is really confused as to what you're doing – this process of presenting your results piecemeal wasn't described in your methods	Amended as above
• Table 4 –<br>o This is the 'nyama' of your multivariable regression. I suggest the other two regression tables aren't needed – once you've described the process in your methods section (as advised above), then this is really the table that you want to talk about<br>•	The regressions tables have been maintained to demonstrate the variables explored as recommended by another reviewer.
• Research hypothesis and test result findings – remove this section completely (and note – you don't describe Fishers and Poisson in your methods, but Mantel log-rank and Cox – details matter!)	Table removed.
discussion/Conclusion:<br>• More is not better: You need to decide what your absolutely critical takeaway message is for you paper – you've currently got multiple messages in here and nothing comes	My understanding of the decision is discussing all the findings of the study, and seeing how they relate to findings elsewhere, then identifying the key takeaway points (conclusions) and giving recommendations of your findings. This is what I attempted to do in this

out clearly as the main point<br>• This should be about 2 pages long – most of what you have in here now actually belongs in your introduction section, prime examples being the paragraphs which start:<br>○ “Partner involvement…”<br>○ “A retrospective study in Tamil…”<br>○ “In rural Kenya…”<br>• Don’t repeat study definitions in your discussion section<br>• The second half of paragraph 1 is written nicely – you’ve clearly reframed your findings and compared them to regional findings – you don’t need to add all the CI’s though – you can just say “92% in Rwanda and 73% in Cameroon” for example<br>• More is not better: Keep language tight and straightforward - ie – on p20, “Further, add to say that detection of maternal HIV status” becomes “Detection of maternal HIV status”.	particular section.<br><br>Some content revised to ensure proper document flow.<br><br>Definitions removed<br><br>Noted. CI retained as suggested by previous reviewer.<br><br>Noted and actioned.
REVIWER 3	
**<u>General points</u>**<br><br>1. The manuscript retains elements of a dissertation – selected headings, inclusion of textbook formulae, Table 5, the order of headings, amongst others…<br>2. This is a survival *analysis*, and the authors define the event as infant HIV-infection so the appropriate term is **HIV-free survival**. ‘Survival analysis’ implies alive/dead which is not the case and it is inappropriate to describe any variables as factors associated with *survival.* This needs to be amended in the Title and *throughout*.<br>3. The manuscript appears incomplete, particularly parts of the Methods and the Results sections and several Tables. There is *insufficient detail* to	Manuscript edited and unnecessary headings removed.<br><br>Amended and HIV-free survival used throughout the document.<br><br>The Methods section was amended appropriately, and more results added. The flow of the result reworked, spellings corrected and grammar.

provide context to the study and lack of attention to detail in terms of presentation: headings, spelling out acronyms, mixing of tenses, lack of full sentences in the Methods, references referred to as a number in the Discussion, amongst others…<br>4. In the HIV field, there is a move towards using more patient-centred language and avoiding the terms mother-to-child-transmission (use 'Vertical Transmission') and HIV-infected/positive (use 'living with HIV').<br>5. Many of the references are outdated and the results have been super-ceded. The most recent reference is 2019. There is much more contemporary literature available.	Amended to use patient centered language ie mothers living with HIV, or HIV exposed infants instead of HIV positive.<br><br>More updated references have been included in the study.
The document requires complete revision. I suggest you read related peer-reviewed papers and model your manuscript on this presentation, in addition to abiding by PLOS One's style requirements (it is not aligned).	Has been aligned to PLoS one style
<u>Title:</u> As noted above the term 'survival' is misleading as it implies not dying. This is fundamental.	Has been amended to HIV-Free survival as suggested for clarity.
<u>Abstract:</u> This is not detailed enough to provide a comprehensive summary of the study. You should include the outcome variable and main exposure variables. No need to include IRB details in the abstract. The conclusion that factors are associated with 'poor infant survival' is false; these factors are associated with HIV infection.	The outcome variable has been described in the abstract and the main exposure variables discussed in the results section of the abstract. IRB details removed i.e., the IRB number.<br><br>Conclusion amended.
<u>Introduction:</u> The references used to set context are out-dated and need to be updated. To my knowledge, Botswana has	More updated references used

also achieved elimination of vertical transmission of HIV or is close. Not a single acronym is spelled out.	Acronyms spelled out
<u>Methods:</u> This section lacks important details. You should include the vertical transmission guidelines and antenatal and postnatal care guidelines for context. It is essential to include the early infant diagnosis testing protocols.<br><br>Some description of the study population is given but the connection between the description and why the study sites were selected is omitted.<br><br>Many sections are not written in full sentences.<br><br>Why were infants only recruited at 2-6 weeks? Was there any birth testing? If not, then why exclude the youngest group which may have been infected *in utero*?<br><br>The sample size calculation is unclear; what proportion of vertical transmission was expected and why? I cannot determine whether the sample size is appropriate for the information presented.<br><br>From where were the **PCR data** obtained? Lab results, self-report? Were all infants tested as per guidelines?<br><br>**Breastfeeding** is an important variable when considering vertical transmission of HIV after the first couple of weeks. I don't see reference to breast-feeding.<br><br>'Study Validity' section: this does not describe validation of the questionnaire but a pilot study. A validation study is completely different and requires comparison against a gold standard.<br><br>The formulae on pages 9, 10 and 11 are	Guidelines used cited in the infant recruitment and follow up section ie MOH, 2016 (Latest guideline). Guidelines ()PMTCT) also indicated in the introduction<br><br>Reason for selecting study sites included.<br><br>Document proofread and amended<br><br>There was no birth testing during this period and infants were only tested at 6 weeks. Birth testing has only been piloted in parts of Meru and Nyanza region but has not yet been rolled out nationally. The first contact into the EID clinic was done at 2 weeks postnatal.<br>More explanation of how the sample size was determined has been included in the manuscript.<br><br>Information on PCR testing has been added.<br><br>Information on infant breastfeeding was only collected to determine the infant feeding practices but further analysis to test for associations was not done, and hence was not included in the manuscript. There was also no viable way at the time of the study to confirm the feeding options described by the mothers. Additional information on how validity was tested has been included.<br><br>Formulae have been removed.

unnecessary.<br><br>You need to report uncertainty ranges around the central tendency.<br><br><u>Ethics:</u> it is not clear that IRB approval was actually granted.<br><br><u>Limitations</u>: This is an important section and belongs AFTER the Discussion.<br><br>It is suggested here that there may be **HIV exposure misclassification**. In other words, it could not be confirmed that all enrolled infants were actually HIV-exposed as per the inclusion criteria. This is a major limitation and calls into question the results. This should have been dealt with in the study design in that only infants with a confirmed HIV-exposure status should have been enrolled.	I'm not sure what is meant by this, but the results that were not clear have been further elaborated based on other reviewers' comments.<br><br>Wording on ethical clearance redone to show that ethical approval was given to undertake the study.<br><br>Limitations have been moved to the discussion section<br><br>Only infants born to Women Living with HIV were included in the study. Only HIV exposed infants are enrolled for the EID services. This is demonstrated in the title of the manuscript and more clarification of the same done in the inclusion criteria among other sections of the manuscript.
<u>Results:</u> Present the descriptive data first and have a table of baseline characteristics at enrolment.<br><br>How many were screened, over what period etc.<br><br>Present the actual numbers and proportions.<br><br>Death and transfer out are **competing events.** i.e., you cannot become HIV infected if you die or if you transfer out and are not tested. You need to apply a statistical method to account for these competing events.<br><br>Table 1: present actual numbers and proportions with the p-value. The Chi statistic and degree of freedom are not required in the table.<br><br>Tables 2 and 3 seem to be missing most of their data.<br><br>Table 5 doesn't belong in the results section of a manuscript. The 95% CI are very wide.	A descriptive table has been added.<br><br>Narrative describing this has been included in the results section<br><br>Competing events were factored in the modelling process and death was found to be an insignificant factor in the pretext of competing interests. This narrative had not been included in the results but has now been included.<br><br>To the best of my knowledge this is how mantel log rank tests results are presented. I stand corrected.<br><br>All the data for the variables explored have been presented in these tables. However, the clarity of some of the indicators and table titles have been reworked.<br>Table 5 has been removed.

Discussion: It is difficult to comment as the results are not clearly presented.<br><br>Results are referenced for the first time in the discussion.<br><br>It is not usual practice to refer to a reference number in the text. It is preferable (required) to integrate the relevant content from the reference in words in the text.	Discussion has been improved based on other reviewers' comments.<br><br>Not clear on the action point here. However, the results have been discussed in the discussion section and all discussions in the results section removed.<br>Noted and narratives included, where deemed necessary.
References: need updating<br><br>The internet references and reports are incomplete.	Some new references have been included in the manuscript. The manuscript has been proofread for errors.
Prepared by:<br>Dr. Elizabeth Kiilu<br>Tel: +254 721724580<br>Email: elisakiilu09@gmail.com	
