# Supplementary material for: Prognostic factors influencing HIV-free survival among infants enrolled for HIV early infant diagnosis services in selected hospitals in Nairobi County, Kenya
Source: PLoS One. 2023 Oct 4;18(10):e0292427. doi: 10.1371/journal.pone.0292427 (PMC10550113; doi:10.1371/journal.pone.0292427)
Supplement: Attachment — Submitted filename: PONE-D-21-18949_R3_reviewer_25jul_btb.pdf [file pone.0292427.s008.pdf]

PLOS ONE

Prognostic factors influencing HIV-Free survival among infants enrolled for HIV early infant diagnosis services in selected hospitals in Nairobi County, Kenya.

--Manuscript Draft--

**Manuscript Number:**	PONE-D-21-18949R3
**Article Type:**	Research Article
**Full Title:**	Prognostic factors influencing HIV-Free survival among infants enrolled for HIV early infant diagnosis services in selected hospitals in Nairobi County, Kenya.
**Short Title:**	Prognostic factors influencing HIV-free survival among infants enrolled for early infant diagnosis services
**Corresponding Author:**	Elizabeth Mueke Kiilu, Ph.D. in Public Health<br>University of Bradford Faculty of Health Studies<br>Bradford, Bradford UNITED KINGDOM
**Keywords:**	Infant, HIV-free survival, pediatrics, early infant diagnosis, Survival analysis, PMTCT, eMTCT, EID, Vertical transmission, HIV/AIDS
**Abstract:**	Abstract<br><br>Background<br><br>Despite being a preventable disease, pediatric HIV infection continues to be a public health concern due to the morbidity and mortality associated with the disease. Vertical transmission of HIV occurs when a mother living with HIV passes the virus to her baby during pregnancy, childbirth, or breastfeeding. Globally, the vertical transmission rate of HIV is 9% with sub-Saharan Africa accounting for 90% of these infections. In Kenya, the national vertical transmission rates of HIV stood at 11.5% by the end of 2018, with a target to reduce vertical transmission rates to below 5% and 2% in breastfeeding and non-breastfeeding infants respectively, by the end of 2021.<br><br>Objective<br><br>To determine the prognostic factors influencing HIV-free survival among infants enrolled for HIV early infant diagnosis (EID) services in selected hospitals in Nairobi County, Kenya.<br><br>Methods<br><br>A prospective cohort study design was adopted. HIV exposed infants were recruited at six weeks to determine HIV-free survival over 12 months follow up. Simple random sampling was used to select 166 infants and data were collected from the mothers using semi-structured interviewer-administered questionnaires. Log-rank tests were used to test for associations at the bi-variable level while Cox-proportional regression was used to analyze data at the multi-variable level, with the aid of STATA 14 software. Ethical approval was obtained from Kenya Medical Research Institute, Scientific Ethics Review Unit.<br><br>Results<br><br>The overall infant HIV incidence rate over one-year follow-up was 9 cases per 100 person-years (95% CI: 5.465 – 16.290). The failure event was defined as an infant with a positive PCR test during the study period with total failures being 13 (9.41%) over 12

Powered by Editorial Manager® and ProduXion Manager® from Aries Systems Corporation

	months. Prognostic factors associated with poor infant HIV-free survival were young maternal age (18-24 years) and mothers with a recent HIV diagnosis of ≤ 2 years since a positive HIV diagnosis (HR 5.97 CI: 1.20, 29.58) and (HR 6.97 CI: 1.96, 24.76), respectively.<br><br>Conclusion<br><br>Maternal prognostic factors associated with poor infant HIV-free survival were young maternal age (18-24 years) and recent maternal HIV diagnosis of ≤ 2 years since positive HIV diagnosis. The study recommended the development of an intervention package with more rigorous adherence counseling and close monitoring for young mothers, and mothers with recent HIV diagnoses.
**Order of Authors:**	Elizabeth Mueke Kiilu, Ph.D. in Public Health
	Gideon Mutie Kikuvi
	Simon Karanja
	Peter Wanzala
**Response to Reviewers:**	Thank you for the insightful comments that have markedly improved my work. This is very much appreciated.
**Additional Information:**	
**Question**	**Response**
**Financial Disclosure**<br><br>Enter a financial disclosure statement that describes the sources of funding for the work included in this submission. Review the submission guidelines for detailed requirements. View published research articles from *PLOS ONE* for specific examples.<br><br>This statement is required for submission and **will appear in the published article** if the submission is accepted. Please make sure it is accurate.	National Research Fund (NRF)-Government of Kenya, Jomo Kenyatta University of Science, and Technology (JKUAT). The funders had no role in the study design, data collection, analysis, decision to publish, or preparation of the manuscript.

Powered by Editorial Manager® and ProduXion Manager® from Aries Systems Corporation

**Unfunded studies**<br>Enter: *The author(s) received no specific funding for this work.*<br><br>**Funded studies**<br>Enter a statement with the following details:<br>• Initials of the authors who received each award<br>• Grant numbers awarded to each author<br>• The full name of each funder<br>• URL of each funder website<br>• Did the sponsors or funders play any role in the study design, data collection and analysis, decision to publish, or preparation of the manuscript?<br>• **NO** - Include this sentence at the end of your statement: *The funders had no role in study design, data collection and analysis, decision to publish, or preparation of the manuscript.*<br>• **YES** - Specify the role(s) played.<br><br>* typeset	
**Competing Interests**<br><br>Use the instructions below to enter a competing interest statement for this submission. On behalf of all authors, disclose any competing interests that could be perceived to bias this work—acknowledging all financial support and any other relevant financial or non-financial competing interests.<br><br>This statement is required for submission and **will appear in the published article** if the submission is accepted. Please make sure it is accurate and that any funding sources listed in your Funding Information later in the submission form are also declared in your Financial Disclosure statement.<br><br>View published research articles from *PLOS ONE* for specific examples.	The authors have declared that no competing interests exist.

**NO authors have competing interests**<br><br>Enter: *The authors have declared that no competing interests exist.*<br><br>**Authors with competing interests**<br><br>Enter competing interest details beginning with this statement:<br><br>*I have read the journal's policy and the authors of this manuscript have the following competing interests: [insert competing interests here]*<br><br>* typeset	
**Ethics Statement**<br><br>Enter an ethics statement for this submission. This statement is required if the study involved:<br><br>• Human participants<br>• Human specimens or tissue<br>• Vertebrate animals or cephalopods<br>• Vertebrate embryos or tissues<br>• Field research<br><br>Write "N/A" if the submission does not require an ethics statement.<br><br>General guidance is provided below. Consult the submission guidelines for detailed instructions. **Make sure that all information entered here is included in the Methods section of the manuscript.**	1. The KEMRI Scientific and Ethics Review Unit (SERU)_Approval Number: KEMRI/SERU/CPHR/002/3525<br>2. Written Consent was obtained<br>3. Benefits and risks explained<br>4. Voluntary participation and withdrawal at any point of the study<br>5. Confidentiality, anonymity and privacy maintained

Powered by Editorial Manager® and ProduXion Manager® from Aries Systems Corporation

**Format for specific study types**<br><br>**Human Subject Research (involving human participants and/or tissue)**<br>• Give the name of the institutional review board or ethics committee that approved the study<br>• Include the approval number and/or a statement indicating approval of this research<br>• Indicate the form of consent obtained (written/oral) or the reason that consent was not obtained (e.g. the data were analyzed anonymously)<br><br>**Animal Research (involving vertebrate animals, embryos or tissues)**<br>• Provide the name of the Institutional Animal Care and Use Committee (IACUC) or other relevant ethics board that reviewed the study protocol, and indicate whether they approved this research or granted a formal waiver of ethical approval<br>• Include an approval number if one was obtained<br>• If the study involved *non-human primates*, add *additional details* about animal welfare and steps taken to ameliorate suffering<br>• If anesthesia, euthanasia, or any kind of animal sacrifice is part of the study, include briefly which substances and/or methods were applied<br><br>**Field Research**<br><br>Include the following details if this study involves the collection of plant, animal, or other materials from a natural setting:<br>• Field permit number<br>• Name of the institution or relevant body that granted permission	
**Data Availability**<br><br>Authors are required to make all data underlying the findings described fully available, without restriction, and from the time of publication. PLOS allows rare exceptions to address legal and ethical concerns. See the PLOS Data Policy and FAQ for detailed information.	No - some restrictions will apply

A Data Availability Statement describing where the data can be found is required at submission. Your answers to this question constitute the Data Availability Statement and **will be published in the article**, if accepted.<br><br>**Important:** Stating 'data available on request from the author' is not sufficient. If your data are only available upon request, select 'No' for the first question and explain your exceptional situation in the text box.<br><br>Do the authors confirm that all data underlying the findings described in their manuscript are fully available without restriction?	
**Describe where the data may be found in full sentences. If you are copying our sample text, replace any instances of XXX with the appropriate details.**<br><br>• If the data are **held or will be held in a public repository**, include URLs, accession numbers or DOIs. If this information will only be available after acceptance, indicate this by ticking the box below. For example: *All XXX files are available from the XXX database (accession number(s) XXX, XXX.).*<br>• If the data are all contained **within the manuscript and/or Supporting Information files**, enter the following: *All relevant data are within the manuscript and its Supporting Information files.*<br>• If neither of these applies but you are able to provide **details of access elsewhere**, with or without limitations, please do so. For example:<br><br>*Data cannot be shared publicly because of [XXX]. Data are available from the XXX Institutional Data Access / Ethics Committee (contact via XXX) for researchers who meet the criteria for access to confidential data.*<br><br>*The data underlying the results presented in the study are available from (include the name of the third party*	The minimal underlying data set had been provided as part of supporting files for reviewer access. The data set has been de-identified, however, the data set remains part of the Government of Kenya, Ministry of Health property, and permission to share it publicly needs to be sought from the director general using the following contacts:<br>Dr. Patrick Amoth<br>Ag. Director General for Health at Ministry of Health<br>Ministry of Health,<br>Afya House, Cathedral Road,<br>P.O. Box:30016–00100, Nairobi, Kenya.<br>Email: dghealth2019@gmail.com

and contact information or URL).<br>• This text is appropriate if the data are owned by a third party and authors do not have permission to share the data.<br><br>* typeset	
Additional data availability information:	

Powered by Editorial Manager® and ProduXion Manager® from Aries Systems Corporation

Manuscript
Click here to access/download;Manuscript;Manuscript.docx

Prognostic factors influencing HIV-Free survival among infants enrolled for HIV early infant diagnosis services in selected hospitals in Nairobi County, Kenya

Elizabeth Mueke Kiilu [1*], Simon Karanja, [2¶], Gideon Kikuvi [3¶], Peter Wanzala [4&]

[1]School of Health Science, University of Bradford, United Kingdom

[2]School of Public Health, Jomo Kenyatta University of Agriculture and Technology, Nairobi, Kenya

[3]School of Public Health, Jomo Kenyatta University of Agriculture and Technology, Nairobi, Kenya

[4]Center of Public Health Research, Kenya Medical Research Institute Nairobi, Kenya

[#a] Current address: School of Public Health, Jomo Kenyatta University of Agriculture, and Technology PO BOX 62000 - 00200, Nairobi, Kenya

[*]Corresponding author: Elizabeth Mueke Kiilu

E-mail: elisakiilu09@gmail.com/elizabeth.kiilu@jkuat.ac.ke

[¶]These authors contributed equally to this work

[&] These authors contributed equally to this work

Abstract

Background

Despite being a preventable disease, pediatric HIV infection continues to be a public health concern due to the morbidity and mortality associated with the disease. Vertical transmission of HIV occurs when a mother living with HIV passes the virus to her baby during pregnancy, childbirth, or breastfeeding. Globally, the vertical transmission rate of HIV is 9% with sub-Saharan Africa accounting for 90% of these infections. In Kenya, the national vertical transmission rates of HIV stood at 11.5% by the end of 2018, with a target to reduce vertical transmission rates to below 5% and 2% in breastfeeding and non-breastfeeding infants respectively, by the end of 2021.

Objective

To determine the prognostic factors influencing HIV-free survival among infants enrolled for HIV early infant diagnosis (EID) services in selected hospitals in Nairobi County, Kenya.

Methods

A prospective cohort study design was adopted. HIV exposed infants were recruited at six weeks to determine HIV-free survival over 12 months follow up. Simple random sampling was used to select 166 infants and data were collected from the mothers using semi-structured interviewer-administered questionnaires. Log-rank tests were used to test for associations at the bi-variable level while Cox-proportional regression was used to analyze data at the multi-variable level, with the aid of STATA 14 software. Ethical approval was obtained from Kenya Medical Research Institute, Scientific Ethics Review Unit.

Results

The overall infant HIV incidence rate over one-year follow-up was 9 cases per 100 person-years (95% CI: 5.465 – 16.290). The failure event was defined as an infant with a positive PCR test during the study period with total failures being 13 (9.41%) over 12 months. Prognostic factors associated with poor infant HIV-free survival were young maternal age (18-24 years) and mothers with a recent HIV diagnosis of ≤ 2 years since a positive HIV diagnosis (HR 5.97 CI: 1.20, 29.58) and (HR 6.97 CI: 1.96, 24.76), respectively.

Conclusion

Maternal prognostic factors associated with poor infant HIV-free survival were young maternal age (18-24 years) and recent maternal HIV diagnosis of ≤ 2 years since positive HIV diagnosis. The study recommended the development of an intervention package with more rigorous adherence counseling and close monitoring for young mothers, and mothers with recent HIV diagnoses.

Introduction

Globally, vertical transmission of Human Immunodeficiency Virus (HIV) accounts for more than 200,000 new infections in children yearly with 90% of these infections happening in Sub-Saharan Africa. This has necessitated the World Health Organization (WHO) to create a task force to spearhead the elimination of mother-to-child transmission (eMTCT) of HIV and syphilis by 2030 and, together with other development partners formed a global criterion to validate the elimination of vertical transmission of HIV [1]. In 2017, Cuba became the first country (among the low and middle-income countries) to be validated as having successfully eliminated the vertical transmission of HIV and Syphilis. Other countries that achieved these milestones include the Republic of Moldova, Thailand, Belarus, Armenia, and recently in 2022, Oman has been validated by WHO as having eliminated vertical transmission of HIV [1, 2]. These accomplishments of elimination of vertical transmission of HIV are due to the integration of MCH with sexual, reproductive health, and HIV services alongside the engagement of communities and outreach initiatives to reach marginalized populations [1].

In Kenya, the elimination of vertical transmission of HIV and early infant diagnosis (EID) cascades of care/guidelines are contained in the Kenya framework for eMTCT of

HIV and syphilis, Guidelines on Use of antiretroviral (ARV) drugs for preventing HIV infection in Kenya, among other County-specific guidelines [3]. These guidelines stipulate care and treatment modalities for mothers living with HIV during pregnancy, delivery up to cessation of breastfeeding, and infant care up to 18 months or up to the end of the period. In summary, the guidelines stipulate that testing for HIV in HIV exposed infants be done at 4-6 weeks of life or at the earliest opportunity thereafter. All infants should be initiated on prophylaxis at birth and mothers must continue with their highly active antiretroviral therapy (HAART) regimen (first or second-line ART regimens) throughout the pregnancy and breastfeeding periods and continue taking ART for life.

HIV-free survival was proposed as the gold standard for the measurement of prevention of vertical transmission of HIV and EID program effectiveness based on maternal-related factors, child factors and health system-related factors [1].

Infant HIV-free survival in HIV-exposed infants is one of the most important measures of prevention of vertical transmission of HIV and EID program effectiveness, which not only acknowledges the availability, and effectiveness of therapeutic interventions but also the competing threats to infant HIV-free survival [3]. Survivorship in this study was defined in terms of infant HIV status at the end of 12 months whereby the infant is HIV-free between 6 weeks (when the first Polymerase Chain Reaction-PCR test is undertaken) and 12 months of age.

Methods

The study adopted a prospective cohort study design approach whereby mother-infant pairs were followed up for 12 months. The study was conducted in the following public healthcare facilities: Mathare North Health Centre, Mbagathi County Hospital, and Kibera South Health Centre in Nairobi County within the catchment area of Kibra and Mathare informal settlements. These study sites were chosen because approximately 60-70% of urban dwellers in Kenya are believed to be living in the slum area [4, 5] with a 12% prevalence of HIV compared to a 5% prevalence among non-slum dwellers. The study population was infants born to mothers living with HIV enrolled for EID services at Mathare North Health Center, Mbagathi County Hospital, and Kibera South Health Center in Nairobi County.

A total of 166 participants were selected using simple random sampling with the sampling frame being the post-natal registers in the three identified healthcare facilities. The formula for calculating longitudinal studies was used as shown below:

n = [□∝√1+□1□∗ (1−□∗)+□□√□1 (1−□1)/□+□2(1−□2)]2

(□1−□22)

Where:

P_1 = 0.45 (Proportion of HIV positive infants born to mothers on ART in 2015- GOK (Government of Kenya), 2016)

P_2 = 0.14 (Proportion of HIV positive infants born to mothers not on ART in 2015 - GOK, 2016)

p1 = response proportion in group 1 (q1 = 1 − p1)

p2 = response proportion in group 2 (q2 = 1 − p2)

$\bar{p} = (p1 + p2)/2$

$\bar{q} = 1 - \bar{p} = 1.96$

The study was powered at 0.80 with the level of significance set at 0.05%. A sample size of 166 respondents (after adjusting for 40% loss to follow-up) was selected, with 163 (98.2%) respondents participating in the study. The distribution of infants selected in the three facilities was based on the number of mothers living with HIV that attended the PMTCT clinics in the selected healthcare facilities between October 2015 and October 2016. They were then selected proportionately based on the number of mothers living with HIV that attended the PMTCT clinics in each of these three healthcare facilities. In Mathare North health center (MNHC) 80 infants were selected, in Kibera South health center (KSHC) KSHC 60 infants were selected, while in Mbagathi County hospital (MDH) 26 infants were selected. This gave a total of 166 infants selected to participate in the study.

Simple random sampling technique was used to select the study participants using tables of random numbers. The postnatal register was used as a sampling frame from which participants who met the inclusion criteria were selected. Once selected from the postnatal register, the mother-infant pairs were interviewed as they came for EID services from 2 weeks to 6 weeks after birth. The inclusion criteria constituted: HIV-exposed infants at the age of 2 weeks post-delivery up to ≤ 6 weeks of age, HIV-exposed infants whose mothers were registered in the Comprehensive Care Clinic (CCC) of the selected facilities (CCC is where all clients living with HIV seek care and treatment), and HIV-

exposed infants whose mothers agreed to participate in the study. The exclusion criteria were HIV-exposed infants whose mothers had visited the CCCs in the selected hospitals but were in transit and, HIV-exposed infants whose mothers were newly transferred from other healthcare facilities (had been in the CCC for less than one year prior to the study onset). These mother-infant pairs were excluded from the study as there were no records to corroborate the history obtained from the mothers.

Recruitment of infants into the study was conducted 2 weeks after delivery. The infants were followed up for 12 months to assess their HIV status. Infants' follow-up was done in their first year of life as morbidity and mortality among HIV exposed infants is highest within this period. Infant follow-up was scheduled to coincide with the Kenya Expanded Program for Immunization (KEPI) timetable at 6, 10, 14 weeks, and 12 months. The infants were also followed up at 6 months as per the Government of Kenya (GOK) EID schedule (GOK, 2016).

Results for the HIV virological testing and other health parameters were collected from HIV-exposed infants at 6 weeks, 6 months, and 12 months. The PCR results were obtained from the PCR testing laboratories as documented in the EID registers of the respective healthcare facilities. A scheduled visit was considered a delayed visit if the mother failed to return within one week of the scheduled appointment. The EID process from birth to the announcement of the infants' results to the mothers was considered to be ‘‘complete’’ if the PCR tests were performed at the scheduled time points and the final PCR results were provided to mothers at 12 months of the infant's age. The vital status of

infants who did not return for a follow-up visit was determined by phone calls at 12 months, and an oral interview was conducted to establish reasons for loss to follow up.

A semi-structured interviewer-administered questionnaire was adopted and customized from the World Health Organization data collection and analysis tools for HIV. The semi-structured interviewer-administered questionnaire was used to collect information from the mothers living with HIV by the researcher. The researcher was assisted by six research assistants. Data collection was done at scheduled time points at 6, 10, and 14 weeks, 6 months, and 12 months in the selected healthcare facilities. The semi-structured interviewer-administered questionnaires collected the following data: socio-demographic and socio-economic data of the mother, disclosure of HIV status, maternal characteristics during while attending the prevention of vertical transmission sessions, infant feeding practices, and ART adherence.

A data abstraction tool was used to collect quantitative data from the mother’s health records. The data abstraction tool collected information from the mothers' CCC follow-up file on maternal body mass index (BMI), viral load count, HIV staging, ART adherence, ART regimen, and presence of opportunistic and co-infections in the mother. The collected data was presented in tables and charts. To test the validity of the instrument, a pretest of the data collection tools was done at Langata Health Centre, which has a system almost similar to the selected hospitals and serves the Kibera and Mathare catchment population. In total, 10% (16) of the questionnaires were used to assess the reliability of the questionnaires. The data collected was analyzed, summarized and the findings disseminated to the providers at Langata Health Centre. The reliability of the

data collection tool was tested using Cronbach's alpha technique whereby reliability indexes obtained in the study were all above 0.7 and deemed acceptable.

Data cleaning, coding, and analysis were done using STATA Version 14. Descriptive statistics were used to explore and summarize the data. The HIV-free survival function (the outcome of the study) was assessed using Kaplan- Meier (gave a summary of the HIV-free Survival function and Hazard functions) and Mantel Log-rank was used to test for association between the identified variables. Cox-proportional Hazard test at the multi-variable level tested for the relative risk of the event occurring (infants converting from HIV negative to HIV positive).

The Z values and corresponding p-values and Hazard ratios and their corresponding confidence intervals were reported. Cumulative incidence function was used to model for possible competing interests i.e., death and transfer outs. Finally, the proportional hazard assumption using Schoenfield's residual was used to assess the best-fit model by ensuring that the Cox-hazard assumption was met. The level of significance was set at $P < 0.05$ at 95% confidence interval. The study was powered at 80%. All significant variables at p= <0.1 were used in the forward and backward modeling process to determine the best model using only two prognostic variables. Modeling to choose the most appropriate model from several other competing models was done using Akaike's Information Criteria (AIC). The Schoenfeld residual test was used to assess if the Cox-hazard proportion assumption was met for each model created.

The researcher received ethical approval to conduct the study from the Kenya Medical Research Institute (KEMRI) Scientific Ethics Review Unit (SERU),

KEMRI/SERU/CPHR/002/3525. Clearance to carry out the study was given by the Board of Postgraduate Studies (BPS) of Jomo Kenyatta University of Agriculture, Science, and Technology (JKUAT). Permission to carry out the study was also granted by the Nairobi County Ministry of Health and the Medical Superintendent of the facilities where the study was conducted. Informed consent was obtained from the mothers, and the benefits and risks of the study were explained to them too. Confidentiality, privacy, and anonymity were maintained, and participants were free to withdraw from the study without implications for the mother-infant pairs.

Results

All HIV exposed infants recruited into the study were screened for HIV using the PCR test. A total of 166 mother-infant pairs were recruited for the study and 163 (98.2%) mother-infant pairs participated at the beginning of the study (6 weeks). The 3 (1.8%) mother-infant pairs were censored from the study before 6 weeks of age as follows: at two and a half weeks one infant transferred out of Mathare North Health Center, at three weeks one infant died and at five weeks another one infant died. At six months, 22 mother-infant pairs were LTFU, and 10 more mothers were LTFU at 12 months.

Most 75(45%) of the mothers were aged between 25-34 years while the rest were aged between 18-24 years and 35-44 years, 34(20%) and 57 (34.3%) at recruitment. Slightly above half 93 (56.0%) of the participants had a primary level of education or below. The majority of the mothers were married 141 (84.9%) at recruitment and 113 (86.3%) at the endpoint. Most of the mothers had informal employment 108 (65.1%) at recruitment and

79 (60.3%) at endpoint, with slightly above half of the mothers 95 (57.2%) with a household income that ranged between Ksh. 6001-12000 (Table 1).

Table 1. Socio-demographic and socio-economic characteristics of HIV positive mothers

Maternal characteristics	Time-points	
	Recruitment (6 weeks)	Endpoint (12 months)
	Frequency (%) (n=163)	Frequency (%) (n=131)
**Age in completed years**		
18-24	34(20.5)	27(20.6)
25-34	75(45.2)	58(44.3)
35-44	57(34.3)	46(35.1)
**Highest Level of Education**		
≤Primary	93(56.0)	75(57.3)
≥Secondary	73(44.0)	56(42.8)
**No. of persons living in the household**		
2-5	121(72.9)	88(67.2)
6-8	45(27.1)	43(32.8)
**Respondent marital status**		
Single	25(15.1)	18(12.7)
Married	141(84.9)	113(86.3)
**Employment status**		
Formal	27(16.3)	21(16.0)
Informal	108(65.1)	79(60.3)
Unemployed	31(18.6)	31(23.7)
**Monthly income (Ksh.)**		
≤6000	34(20.5)	37(28.2)
6001-12000	95(57.2)	67(51.2)
≥ 12001-18000	37(22.3)	27(20.6)

The incidence rate was 9 cases per 100 person-years (CI 5.43, 6.10) was obtained at the end of the 12-month study period. The failure event was defined as an infant with a positive PCR test during the study period with total failures being 13 (9.41%) at the end of the study time point (12 months). Slightly above half 85 (52.15%) of the infants had timely recruitment (≤ 6 weeks) for EID service delivery.

Kaplan-Meier HIV-free survival estimate curve over a one-year follow-up

Kaplan–Meier estimate curves showed that the probability HIV-free survival of an infant over a 12-month follow-up period. (Fig 1).

Fig 1: Kaplan-Meier infant HIV-free survival estimate over a one-year follow-up period.

Kaplan-Meier infant failure estimate curve over a one-year follow-up

Kaplan-Meier failure estimate curves showed that the probability of HIV vertical transmission of an infant over a 12-month follow-up period was 0.09350 cases per person-year which translated to 9 cases per 100 person-years (95% CI 5.43, 16.10). Thirteen infants turned HIV positive over the 12-month follow-up period (Fig 2).

Fig 2: Kaplan-Meier infant hazard failure estimate over a one-year follow-up period

Mantel log-rank HIV-free survival estimates for HIV-exposed infants over a one-year follow-up period

The Mantel Log-rank test was used to determine factors associated with HIV-free survival at univariate level. This is as shown in (Table 2). At recruitment (6weeks) 161 (97.0%) mothers reported that they were exclusively breastfeeding their infants while 3 (1.8%) did mixed feeding and 2 (1.2%) were on formula milk. Only 75 (53.2%) of the infants were exclusively breastfed for six months, while 64 (45.4%) were mixed fed and 2 (1.4%) were on formula milk for the first six months of their lives. Cross tabulations for infant feeding practices were not undertaken as there was no way to corroborate the mothers report on the infant feeding practices during the study period.

All the infants were put on prophylaxis from birth up to 6 weeks. At 6 weeks, their HIV status was determined using a PCR test for further care and treatment. At 6months and 12 months, PCR tests were repeated on the infants.

Table 2. Maternal factors associated with infant HIV-free survival.

**Prognostic factors (n = 131)**	**Chi-square test**	**p-value**	**DF**
**Maternal Socio-demographic and economic characteristics**			
Age in completed years	7.39	**0.025**	2
Highest Level of Education	0.12	0.733	1
No. of persons living in the household	0.04	0.841	1
Respondent marital status	3.41	0.065	1
Employment status	23.93	**<0.001**	2
Household Monthly income (Ksh)	17.98	**0.001**	2
**Maternal characteristics during PMTCT clinic visits**			
Year Confirmed Positive	11.86	**<0.001**	1
Mode of Delivery	0.18	0.668	1
Given ART in pregnancy	12.66	**<0.001**	1
Stage ART administered in pregnancy	20.66	**<0.001**	2
Gestation of pregnancy at 1st PMTCT visit	0.16	0.923	2
No of PMTCT clinic visits	19.58	**<0.001**	2
**Maternal disclosure status**			
Have a partner	0.24	0.627	1
Disclosed status to the partner	9.64	**0.002**	1
Know Partner's HIV status	10.80	**0.001**	1
Spouse HIV Status	0.17	0.678	1
**Maternal characteristics at 12 Months**			
ART adherence (Morisky Score)	33.71	**<0.001**	2
Maternal HIV Staging	26.94	**<0.001**	1
ART Regimen	81.58	**<0.001**	1
Viral Load (VL)	42.24	**<0.001**	2
Maternal BMI	17.23	**0.001**	3
**Timeliness into EID enrollment at 6 weeks (recruitment) among exposed infants**			
Timeliness at 6 weeks (recruitment)	5.22	**0.022**	1

Cox Regression analysis for socio-economic and demographic factors showing HR and AHR over 12 months follow-up period

Table 3 gives a summary of the Cox regression analysis for socio-demographic and socio-economic factors, maternal factors during the attendance of prevention of vertical transmission clinics (PMTCT clinics) and the corresponding hazard ratios and adjusted hazard ratios.

Table 3. Cox Regression analysis of socio-economic and demographic factors, showing HR and AHR over 12 months follow-up

**Time point (n= 166)**	**Infant HIV-free survival over the 12-month follow-up period**	
**Maternal characteristics**	**HR (95%CI)**	**AHR (95%CI)**
**Age in years**		
35-44 *(Ref)*	1.00	
18-24	**5.97(1.20, 29.58)**	
25-34	1.96 (0.38, 10.13)	
**Employment**		
Unemployed (*Ref)*	1.00	
Formal	0.14 (0.02, 1.10)	
Informal	**0.07 (0.02, 0.31)**	
**Monthly Household Income**		
≤ 6000 *(Ref)*	1.00	
6001-12000	**0.10 (0.02, 0.44)**	
≥12001-18000	**0.12 (0.02, 0.10)**	
**Year confirmed +ve**		
> 2 years since positive HIV diagnosis	1.00	
≤ 2 years since positive HIV diagnosis	**6.97(1.96, 24.76)**	

Cox Regression analysis for disclosure status and prevention of vertical transmission factors showing HR and AHR over 12 months follow-up.

Table 4 gives a summary of the Cox regression analysis for maternal disclosure status and maternal factors at 12 months and the corresponding hazard ratios and adjusted hazard ratios.

Table 4. Cox Regression analysis for disclosure status and prevention of vertical transmission factors showing HR and ARH over 12 months follow-up period

Time point (n= 166)	Infant Survival over 12M follow-up period	
**Maternal characteristics**	**HR (95%CI)**	**AHR (95%CI)**
**Disclosed status to partner**		
Yes *(Ref)*	1.00	
No	**5.10 (1.65, 15.82)**	
**Know partner's HIV status**		
Yes *(Ref)*	1.00	1.00
No	**5.87 (1.80, 19.16)**	**4.56(1.27, 16.45)**
**ART Adherence**		
Poor *(Ref)*	1.00	
Good	**0.05 (0.01, 0.23)**	
Inadequate	**0.14 (0.04, 0.50)**	
**HIV Staging**		
I *(Ref)*	1.00	
II	**17.84 (3.94, 80.89)**	
**Viral Load (VL)**		
High VL *(Ref)*	1.00	
...Undetectable VL	**0.02 (0.01, 0.17)**	
...Low VL	**0.12 (0.02,0.90)**	
**...ART Regimen**		
...Second-line *(Ref)*	1.00	1.00
...First-line	**0.03 (0.01, 0.10)**	**0.03 (0.01, 0.08)**
**...Given ART during pregnancy**		
...No *(Ref)*	1.00	
...Yes	**0.11 (0.02, 0.52)**	
**...Stage ART admin in Pregnancy**		
...Third Trimester *(Ref)*	1.00	
...Frist Trimester	**0.09 (0.02, 0.31)**	
...Second Trimester	0.38 (0.10, 1.45)	
**...No. of PMTCT visits attended**		
...2 *(Ref)*	1.00	
...≤1	0.86 (0.23, 3.26)	
...≥3	**0.12 (0.03, 0.40)**	**0.04 (0.01, 0.25)**
**...Maternal BMI**		
…Normal *(Ref)*	1.00	
...Underweight	**6.29 (1.93, 20.47)**	
...Overweight	0.64 (0.12, 3.26)	
...Obese	0.80 (0.09, 6.90)	
**...Timeliness into EID enrollment**		
...Timely (≤6weeks) *(Ref)*	1.00	
...Delayed (>6weeks)	**4.00 (1.12, 14.32)**	

Final model of maternal factors influencing infant HIV-free survival

All significant variables at p= <0.1 were included in the modelling process to determine the best model using only two prognostic variables. Cumulative incidence function was used to model for possible competing interests i.e., death and transfer outs. In the final HIV-free survival model, mothers on first-line ART regimen and had attended more than three PMTCT clinic visits and, knew their partner's HIV status had lower hazard ratios of experiencing infant HIV vertical transmission, relative to mothers that were on the second-line ART regimen, had attended fewer antenatal clinic (ANC) visits and did not know their partners HIV status respectively (Table 5).

Table 5. Final model of maternal factors influencing HIV-free survival of infants

Maternal characteristics	Hazard Ratio	Robust Std. Err.	z	P>z	[95%C. I] Lower	Upper	Prob > Chi2
							**<0.001**
**ART Regimen**							
Second-line *(Ref)*	1.000						
First-line	0.028	0.016	-6.3	**<0.001**	0.0091	0.083	
**No. of PMTCT clinic visits**							
2 *(Ref)*	1.000						
≤1	0.536	0.485	-0.6	0.491	0.091	3.162	
≥3	0.046	0.040	-3.5	**<0.001**	0.008	0.255	
**Know partner's HIV status**							
Yes *(Ref)*	1.000						
No	4.564	2.9853	2.32	**0.020**	1.266	16.448	
**Summary of final HIV-free survival model at 12 months**							
**Model p-value**	**n**	**Log-Likelihood**	**AIC**	**BIC**	**Schofield residual test**		**DF**
<0.001	131	-30.15	68.32	79.62	0.86		4

Discussion

Infant HIV-free survival in HIV-exposed infants is one of the most important measures of PMTCT and EID programs effectiveness, which not only acknowledges the availability, and effectiveness of therapeutic interventions but also the competing threats to infant HIV-free survival [3]. In the current study, HIV-free survival among the HIV-exposed infants was estimated at 90.7% (with a total person-time of 139.02 days, 95% CI 5.43, 16.10) for the infants aged between 6 weeks and 12 months a finding similar to that in Rwanda 91.9% [6] but lower than what was observed in Zambia 96.3% (95% CI: 94.8, 97.4%) [7], and much higher than in Cameroon (72.6%; 95% CI: 62.3, 80.5) and South Africa (77.7%; 95% CI: 72.5, 82.1) [8].

In a similar longitudinal study undertaken in Malawi [9] the vertical transmission rate of HIV was 4.9% (95% CI 3.7, 6.4%), much lower than what was observed in the current study i.e., 9.41%. The authors attributed poor infant HIV-free survival to factors similar to those seen in the current study i.e., mothers not disclosing their HIV status to their partners, poor maternal ART adherence and late maternal ART initiation. South Africa [10] had a similar vertical transmission rate of HIV as that seen in Malawi [9] but much lower in than in the current study. However, reasons for poor infant HIV-free survival in the South African study [10] differed from what was seen in Malawi and Kenya and was attributed to unknown maternal CD4-cell-count undocumented maternal HIV status and exclusive or mixed breastfeeding [10].

Maternal age influenced infant HIV-free survival over the 12 months follow-up period whereby younger mothers aged between 18-24 years had a higher hazard ratio (5.97) of infant HIV vertical transmission relative to their older counterparts 36-47 years, a finding similar to what was observed in Rwanda, Malawi Swaziland, and Kinshasa [7, 9, 11, 12] respectively whereby young maternal age was associated with an increased risk of infant HIV vertical transmission. This however differed from findings observed in South Africa whereby young maternal age was not associated with infant HIV- free survival [13].

Unemployment and low household incomes were facilitators of poor infant HIV-free survival in the current study. Poverty increases the chances of infant HIV vertical transmission [13]. Diagnosing mothers early before pregnancy and putting them on ART improves infant HIV-free survival and reduces infant chances of HIV infection. This was supported by [14] where the authors found that failure to diagnose HIV infection during early pregnancy was a primary reason for missing both prevention of vertical transmission of HIV (PMTCT) and EID interventions contributing disproportionately to new pediatric infections.

In the current study, mothers who had a recent HIV positive diagnosis (within two years of HIV diagnosis) had a higher hazard ratio (HR= 6.97) of infant HIV vertical transmission relative to mothers that had lived with HIV for a period of more than two years. Diagnosis during pregnancy poses a challenge for disclosure and sometimes drug compliance and consequently increases the risk of infant HIV vertical transmission [15].

Mothers who received ART during pregnancy had a lower hazard (HR=0.11) of infant HIV vertical transmission relative to mothers that had not received ART during

pregnancy a finding similar to Rwandan and Ethiopian studies [6, 16] respectively. Attending more than three PMTCT clinic visits had a lower hazard ratio (HR= 0.12) of infant HIV vertical transmission relative to mothers who had attended lesser PMTCT clinic visits. In a retrospective study conducted in Zambia [17] mothers that received antenatal care yielded an estimated 89.8% (95% CI: 86.8, 92.2) infant HIV-free survival, lower vertical transmission of HIV rate and higher retention rates to the PMTCT clinics.

A study in Lodwar, Kenya [18] found that mothers who attended more than one PMTCT clinic had higher odds of 2.78 (CI: 1.25, 6.17) of administering prophylaxis to their infants relative to mothers who had attended less than one PMTCT clinic visit. In the current study, non-disclosure of HIV status and not knowing a partner's HIV status had higher hazard ratio (HR= 5.10) and (HR= 5.87) respectively of infant HIV vertical transmission relative to mothers that had disclosed their status and knew their partners HIV status. Male partner involvement through participation in ANC attendance has been associated with enhanced uptake of prevention of vertical transmission of HIV services specifically, improvements in HIV testing and partner disclosure, ART adherence, and improved infant feeding strategies [2]. A study conducted in Papua New Guinea revealed lower infant HIV-free survival (in terms of infant death and infant HIV vertical transmission) and loss to follow-up in mothers that had reported untested or unknown partner HIV status [19]. However, in Malawi, the findings revealed that male partner involvement was not associated with lower risk of infant HIV vertical transmission [20]. In South Africa male partners provided access to medical services whenever the mothers needed them to do so [21].

Male participation during PMTCT clinic attendance improved maternal sexual and reproductive health, reduced stigma and discrimination, and consequently improved infant health outcomes [22]. Non-disclosure of maternal HIV status was associated with an increased risk of vertical transmission of HIV to infants and high maternal viral load [23]. Additionally male involvement increased the incidence of HIV testing and counseling and facilitated partner disclosure [24]. Women in sub-Saharan Africa reported that they faced difficulty in negotiating the use of condoms with their male partners despite the fact that they were aware that some of their partners had multiple sexual partners [25]. A few mothers reported that their partners refused to take their ART, and some even refused to go for HIV testing. Negative male partner behaviors put both the mothers and infants at increased risk of high viral loads and vertical transmission [26].

Underweight mothers had a higher hazard ratio (HR= 6.29) of infant HIV vertical transmission relative to mothers with a normal BMI. A study in Rural Kenya demonstrated that poor nutritional status in mothers living with HIV may impair immunity by weakening the epithelial integrity of the body cells, which in turn increases the risk of infant HIV vertical transmission [27].

Close to a half 81 (48.0%) of the infants had late initiation into the EID (> 6 weeks of age) which led to a higher hazard ratio (4.00) of infant HIV vertical transmission relative to mothers who had enrolled their infants for EID at 6 weeks or earlier. The GOK 2016 guidelines [3] recommend all HIV exposed infants should be enrolled for EID any time from birth up to 6 weeks of age. Globally, it is estimated that nearly half of HIV-exposed infants receive an EID test within the first two months but with wide variations in

initiation timeliness in various countries [28]. The success of infant HIV programs is highly dependent on the early identification and enrollment of mother-infant pairs in care and/or treatment [29]. A study conducted in four countries in Asia and Africa (Cambodia, Senegal, Namibia, and Uganda) elicited that the median age of testing among infants referred from PMTCT clinics was approximately 2 months over the life of the program, with less than 50% of these infants being tested well after 2 months of age [30].

This study had several limitations. Firstly, exposure history was from the mother's self-report, and not all the history that was given could be corroborated from the maternal CCC file, Integrated Management of Childhood Illness (IMCI) booklet, and appointment booklets. Where it was not possible to corroborate the mother's self-report, the questionnaires were structured to counter check the responses asked in a different format within the same questionnaire. Secondly, given the phrasing of the questionnaire, it was difficult to determine if the mothers were adherent to ART throughout the pregnancy period, for instance if there were periods of cessation and non-adherence. Adherence, however, was also determined by observation of participant serial viral loads from pregnancy up to one-year post-partum. Due to the small sample size selected, the incidence rate of 9 cases per 100 person-years (95% CI: 5.465 – 16.290) may have been an underestimation of the true vertical transmission of HIV rates. The small sample size gave wide confidence intervals (CIs) (5.5-16.3) which is not as useful for health policy guidance as narrow CI’s.

Another limitation of our study was the lack of detailed data regarding clients that were lost to follow-up since it was not possible to reach them on the phone. Lastly, another

limitation of the study was that data on feeding practices were provided in the manuscript. However bivariable and multivariable analysis on the same was not done because there was no way to corroborate the breastfeeding and other feeding options from the mother's narrative, at the time of the study. A great strength of the study was in the design of the study, whereby a longitudinal study design was applied, and participant outcomes were studied over a period of 12 months using survival analysis.

Conclusion

Scaling up care and treatment for mother-infant pairs more so in resource-limited settings improves overall HIV-free survival among HIV-exposed infants. There is a need to create a special package of care for the younger mothers aged (18-24) and mothers with recent HIV diagnoses (i.e., less than 2 years since HIV positive diagnosis). The special package should have more rigorous adherence and nutritional counseling, with close monitoring of these mother-infant pairs. This should also be accompanied by robust partner HIV status disclosure strategies to enhance partner support for mothers attending the EID services of HIV. These strategies are aimed at improving infant retention and HIV-free survival throughout the EID cascade of care.

Acknowledgements

I wish to express my most heartfelt and sincere gratitude to the almighty God for the good health, protection, and care that He has granted unto me. My sincere gratitude goes out to all the healthcare workers, healthcare facility in-charges, and the study participants for their support and cooperation, and to all those who assisted in any way during the data

collection and analysis period. Special appreciation goes to Prof. Thomas Achia Center for Disease Control (CDC), KEMRI who played a significant role in statistical mentorship.

Author contributions

Conceptualization: Elizabeth M. Kiilu

Data curation: Prof. Achia, Elizabeth M. Kiilu

Formal analysis: Prof. Achia, Elizabeth M. Kiilu, Samuel Mutua, Mary Kamina

Investigation: Elizabeth M. Kiilu

Methodology: Elizabeth M. Kiilu, Simon Karanja, Gideon Kikuvi

Project administration: Elizabeth M. Kiilu

Supervision: Simon Karanja, Gideon Kikuvi, Peter Wanzala

Writing- Original draft: Elizabeth M. Kiilu

Writing- Review & editing: Elizabeth M. Kiilu, Simon Karanja, Gideon Kikuvi, Peter Wanzala

References

1. World Health Organization. Validation of elimination of mother-to-child transmission of HIV, syphilis, and hepatitis B. Geneva. 2023.
2. Alusio A, Bosire R, Gatuguta A, Kiarie J, Nduati R, John-Stewart G, et al. Male Partner Participation in Antenatal Clinic Services is Associated with Improved HIV-free survival Among Infants in Nairobi, Kenya: A Prospective Cohort Study. Journal of Acquired Immune Deficiency Syndrome. 2016;169-174.

3. Government of Kenya. Kenya Framework for the Elimination of Mother-To-Child Transmission of HIV and Syphilis. 2016.
4. UN-Habitat. The state of African Cities 2014: Re-imagining sustainable urban transitions. 2014.
5. Corburn J, Hildebrand C. Slum Sanitation and the Social Determinants of Women's Health in Nairobi, Kenya. Journal of Environmental and Public Health. 2015; 2015:6.
6. Ruton H, Mugwaneza P, Shema N, Lyambabaje A, Bizimana JD, Tsague L, et al. HIV-free survival among nine- to 24-month-old children born to HIV-positive mothers in the Rwandan national PMTCT program: A community-based household survey. Journal of the International AIDS Society. 2012;15(4).
7. Chi BH, Mutale W, Winston J, Phiri W, Price JT, Mwiche A, et al. Infant Human Immunodeficiency Virus-free Survival in the Era of Universal Antiretroviral Therapy for Pregnant and Breastfeeding Women: A Community-based Cohort Study from Rural Zambia. Pediatric Infectious Diseases Journal. 2018; 37(11):1137-1141.
8. Stringer JSA, Stinson K, Tih P, Giganti M, Ekouevi DK, Creek T. et al. Measuring Coverage in MNCH: Population HIV-Free Survival among Children under Two Years of Age in Four African Countries. PLoS Medicine. 2013;10(5).
9. Lettow MV, Barr BA, Oosterhout JJ, Schouten E, Jahn A, Kalua T, et al. The National Evaluation of Malawi's PMTCT Program (NEMAPP) study: 24-month HIV-exposed infant outcomes from a prospective cohort study. HIV Medicine. 2021;23:573-584.

10. Goga AE, Lombard C, Jackson D, Ramokolo V, Ngandu NK, Sherman G, et al. Impact of breastfeeding, maternal antiretroviral treatment, and health service factors on 18-month vertical transmission of HIV and HIV-free survival: results from a nationally representative HIV-exposed infant cohort, South Africa. Journal of Epidemiology & Community Health 2020;74:1069-1077.
11. Chouraya C, Machekano R, Mthethwa S, Lindan K, Mirira M, Kudiabor K, et al. Mother-to-Child Transmission of HIV and HIV-Free Survival in Swaziland: A Community-Based Household Survey. AIDS and Behavior. 2018;22:105-113.
12. Ditekemena J, Matendo R, Colebunders R, Koole O, Bielen G, Nkuna, M. et al. Health Outcomes of Infants in a PMTCT Program in Kinshasa. Antiretroviral Therapy. 2015;14(5): 449-454.
13. Sifunda S, Peltzer K, Rodriguez VJ, Mandell LN, Lee TK, Ramlagan S, et al. (2019) Impact of male partner involvement on mother-to-child transmission of HIV and HIV-free survival among HIV-exposed infants in rural South Africa: Results from a two-phase randomized controlled trial. PLoS ONE. 2019;14(6): e0217467.
14. Abrams EJ, Woldesenbet S, Silva JS, Coovadia A, Black V, Technau KG. et al. Despite Access to Antiretrovirals for Prevention and Treatment High Rates of Mortality Persist Among HIV-infected Infants and Young Children. Pediatric Infectious Diseases Journal. 2017;36(6): 595-601.
15. Agudu NA, Ramadhani HO, Isah C, Erekaha S, Fan-Osuala C, Anaba U. et al. The Impact of Structured Mentor Mother Programs on Presentation for Early Infant Diagnosis Testing in Rural North-Central Nigeria: A Prospective Paired

Cohort Study. Journal of Acquired Immune Deficiency Syndrome. 2017;75: S182-S189.

16. Derebe G, Biadgilign S, Trivelli M, Hundessa G, Robi Z, Gebre-Mariam M. et al. Determinant and outcome of early diagnosis of HIV infection among HIV-exposed infants in southwest Ethiopia. BMC. 2014;7(309).
17. Buzdugan, R., McCoy, S., Watadzaushe, C., Kang, D. M., Petersen, M., Dirawo, J., et al. Evaluating the Impact of Zimbabwe's Prevention of Mother-to-Child HIV Transmission Program: Mother-to-Child HIV Transmission Program: Mother-to-Child HIV Transmission Program: Population-Level Estimates of HIV-Free Infant Survival Pre-Option A. PLoS ONE. 2015;10(8): e0134571
18. Ongaki D, Obonyo M, James R. Factors Affecting Uptake of PMTCT Services, Lodwar County Referral Hospital, Turkana County, Kenya. Association of Providers of AIDS Care. 2019;1(4):1-4.
19. Carmone A, Bomai K, Bongi W, Frank T, Dalepa H, Lofia B, et al. Partner testing, linkage to care, and HIV Free Survival in a Program to Prevent Mother to Child Transmission of HIV in the highlands of Pupa New Guinea. Global Health Action. 2014;(27)7.
20. Kalembo F, Zgambo M, Mulanga A, Yukai D, Ahmanrd, N. Association between male partner involvement and the uptake of PMTCT interventions in Mwanza District, Malawi: A retrospective cohort study. PLoS One. 2013; 8(6): e66517.
21. Adeniyi Vo, Thomson E, Goon D, Ajayi I. Disclosure, stigma if HIV positive child and access to Early Infant Diagnosis in the rural communities of Tambo

District, South Africa: A qualitative exploration of maternal perspective. BMC Pediatrics. 2015;15(98).

22. Yah CS, Tambo E. Why is mother-to-child transmission (MTCT) of HIV a continual threat to newborns in sub-Saharan Africa (sSA). Journal of Infection and Public Health. 2019;12(2019):213-222.
23. Tshweneagae G, Oss V, Mgutshini, T. Disclosure of HIV status to sexual partners by people living with HIV. Curationis. 2015;38(1): E1-6.
24. Imbaya C, Odhiambo-Otieno G, Okello AB. Socio-demographic characteristics, Antenatal Clinic attendance, and PMTCT knowledge of postnatal HIV+ women at an urban public health facility in Nairobi. East and Central Africa Medical Journal. 2014; 1(1):33-40.
25. Thomson K, Telfer B, Opondo AP, Munge J, Ngunga M, Reid, A. Navigating the risks of prevention of mother to child transmission (PMTCT) of HIV services in Kibera, Kenya: Barriers to engaging and remaining in care. PLoS ONE. 2018;13 (1): e0191463
26. Matseke MG, Ruiter RAC, Rodriguez VJ, Peltzer K, Setswe G, et al. Factors Associated with Male Partner Involvement in Programs for the Prevention of Mother-to-Child Transmission of HIV in Rural South Africa. International Journal of Environmental Research and Public Health. 2017; 14(11):1333.
27. Nabwera H.M, Jepkosgei J, Muraya KW, Hassan AS, Molyneux CS, Ali R, et al. What influences feeding decisions for HIV-exposed infants in rural Kenya? International Breastfeeding Journal. 2017;12(31).

28. Essajee S, Bhairavabhotla R, Penazzato M, Kiragu K, Jani I, Carmona S, et al. Scale-up of Early Infant HIV Diagnosis and Improving Access to Pediatric HIV Care in Global Plan Countries: Past and Future Perspectives. Journal of Acquired Immune Deficiency Syndrome. 2017;75(1): S51-S58.
29. Mondi S, Broyles L, Montandon M, Ltoh M, Ochanda B, Langat A, et al. Beyond Early Infant Diagnosis: Changing the Approach to HIV Exposed Infants. Journal of Acquired Immune Deficiency Syndrome. 2018;78(2): S107-S114.
30. Anirban C, Sangeeta T, Robert G, Ndapewa H, Sok P, Charles K, et al. Implementing services for Early Infant Diagnosis (EID) of HIV: a comparative descriptive analysis of national programs in four countries. BioMed Central Public Health. 2011; 11:553.

Supporting information

S1 Fig. 1 Kaplan-Meier infant survival estimate over a one-year follow-up period.

S2 Fig. 2 Kaplan-Meier infant hazard failure estimate over a one-year follow-up period.

Minimal underlying dataset.

Figure two

Click here to access/download;Figure;S2_File.docx

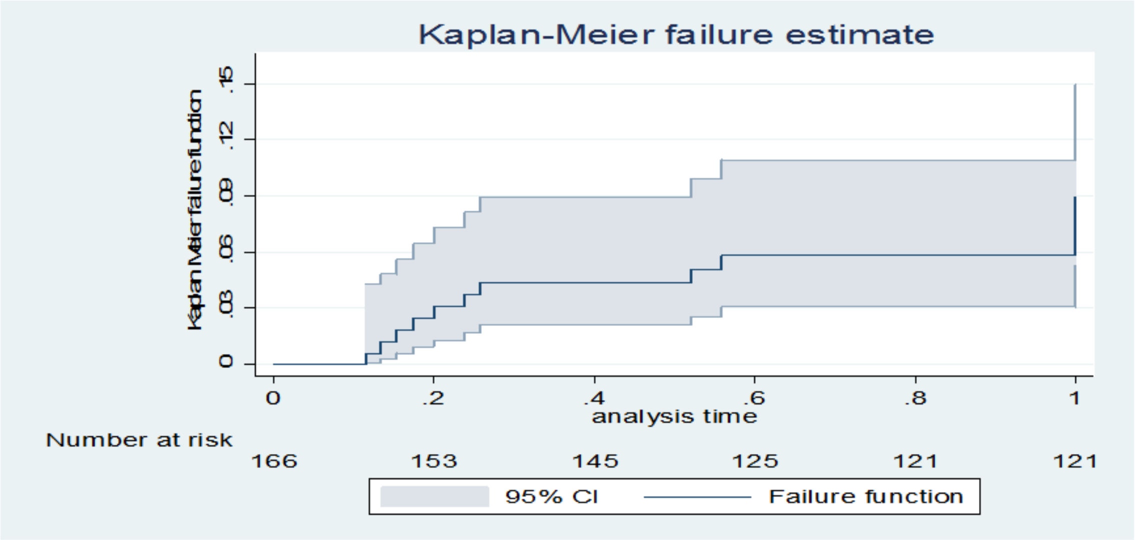

Fig 2: Kaplan-Meier infant hazard failure estimate over a one-year follow-up period

Figure one

Click here to access/download;Figure;S1_File.docx

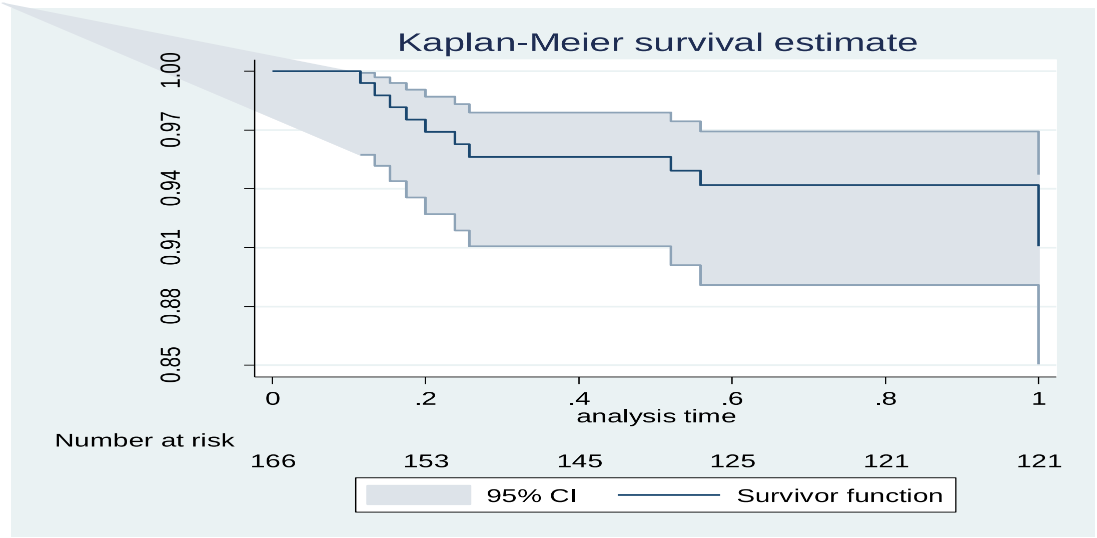

Fig 1: Kaplan-Meier infant survival estimate over a one-year follow-up period

Questionnaire

Click here to access/download
Supporting Information
Questionnaire.pdf

Prognostic factors influencing HIV-Free survival among infants enrolled for HIV early infant diagnosis services in selected hospitals in Nairobi County, Kenya

Elizabeth Mueke Kiilu [1*], Simon Karanja, [2¶], Gideon Kikuvi [3¶], Peter Wanzala [4&]

[1]~~**School of Public Health, Jomo Kenyatta University of Agriculture and Technology, Nairobi, Kenya**~~

[1]**School of Health Science, University of Bradford, United Kingdom**

[2]**School of Public Health, Jomo Kenyatta University of Agriculture and Technology, Nairobi, Kenya**

[3]**School of Public Health, Jomo Kenyatta University of Agriculture and Technology, Nairobi, Kenya**

[4]**Center of Public Health Research, Kenya Medical Research Institute Nairobi, Kenya**

[#a] **Current address: School of Public Health, Jomo Kenyatta University of Agriculture, and Technology PO BOX 62000 - 00200, Nairobi, Kenya**

[*]**Corresponding author:** Elizabeth Mueke Kiilu

E-mail: elisakiilu09@gmail.com/elizabeth.kiilu@jkuat.ac.ke

[¶]These authors contributed equally to this work

[&] These authors contributed equally to this work

Abstract

Background

Despite being a preventable disease, pediatric HIV infection continues to be a public health concern due to the morbidity and mortality associated with the disease. Vertical transmission of HIV occurs when a mother living with HIV passes the virus to her baby during pregnancy, childbirth, or breastfeeding. Globally, the ~~mother-to-child transmission (MTCT)~~vertical transmission rate of HIV ~~rate of HIV~~ is 9% with sub-Saharan Africa accounting for 90% of these infections. In Kenya, the national ~~MTCT~~vertical transmission rates of HIV ~~rates~~ stood at 11.5% by the end of 2018, with a target to reduce ~~MTCT~~vertical transmission rates to below 5% and 2% in breastfeeding and non-breastfeeding infants respectively, by the end of 2021. ~~MTCT of HIV occurs when a mother living with HIV passes the virus to her baby during pregnancy, childbirth, or breastfeeding.~~

Objective

To determine the prognostic factors influencing HIV-free survival among infants enrolled for HIV early infant diagnosis (EID) services in selected hospitals in Nairobi County, Kenya.

Methods

A prospective cohort study design was adopted. HIV exposed infants were recruited at six weeks to determine HIV-free survival over 12 months follow up. Simple random sampling was used to select 166 infants and data were collected from the mothers using semi-structured interviewer-administered questionnaires. Log-rank tests were used to test for associations at the bi-variable level while Cox-proportional regression was used to analyze data at the multi-variable level, with the aid of STATA 14 software. Ethical approval was obtained from Kenya Medical Research Institute, Scientific Ethics Review Unit.

Results

The overall infant HIV incidence rate over one-year follow-up was 9 cases per 100 person-years (95% CI: 5.465 – 16.290). The failure event was defined as an infant with a positive PCR test during the study period with total failures being 13 (9.41%) over 12 months. Prognostic factors associated with poor infant HIV-free survival were young maternal age (18-24 years) and mothers with a recent HIV diagnosis of ≤ 2 years since a positive HIV diagnosis (HR 5.97 CI: 1.20, 29.58) and (HR 6.97 CI: 1.96, 24.76), respectively.

Conclusion

Maternal prognostic factors associated with poor infant HIV-free survival were young maternal age (18-24 years) and recent maternal HIV diagnosis of ≤ 2 years since positive HIV diagnosis. The study recommended the development of an intervention package with more rigorous adherence counseling and close monitoring for young mothers, and mothers with recent HIV diagnoses.

Introduction

Globally, vertical transmission of Human Immunodeficiency Virus (HIV) accounts for more than 200,000 new infections in children yearly with 90% of these infections happening in Sub-Saharan Africa. This has necessitated the World Health Organization (WHO) to create a task force to spearhead the elimination of mother-to-child transmission (eMTCT) of HIV and syphilis by 2030 and, together with other development partners formed a global criterion to validate the elimination of ~~mother-to-child transmission (MTCT)~~ vertical transmission of HIV [1]. In 2017, Cuba became the first country (among the low and middle-income countries) to be validated as having successfully eliminated the ~~MTCT~~ vertical transmission of HIV and Syphilis. Other countries that achieved these milestones include the Republic of Moldova, Thailand, Belarus, Armenia, and recently in 2022, Oman has been validated by WHO as having eliminated ~~MTCT of HIV~~ vertical transmission of HIV [1, 2]. These accomplishments of

eMTCTelimination of vertical transmission of HIV are due to the integration of MCH with sexual, reproductive health, and HIV services alongside the engagement of communities and outreach initiatives to reach marginalized populations [1].

In Kenya, the eMTCTelimination of vertical transmission of HIV and early infant diagnosis (EID) cascades of care/guidelines are contained in the Kenya framework for eMTCT of HIV and syphilis, Guidelines on Use of antiretroviral (ARV) drugs for preventing HIV infection in Kenya, among other County-specific guidelines [3]. These guidelines stipulate care and treatment modalities for mothers living with HIV during pregnancy, delivery up to cessation of breastfeeding, and infant care up to 18 months or up to the end of the period. In summary, the guidelines stipulate that testing for HIV in HIV exposed infants be done at 4-6 weeks of life or at the earliest opportunity thereafter. All infants should be initiated on prophylaxis at birth and mothers must continue with their highly active antiretroviral therapy-HAART regimen (first- or second-line ART regimens) throughout the pregnancy and breastfeeding periods and continue taking ART for life.

HIV-free survival was proposed as the gold standard for the measurement of prevention of mother-to-child (PMTCT) vertical transmission of HIV and EID program effectiveness based on maternal-related factors, child factors and health system-related factors [1].

Infant HIV-free survival in HIV-exposed infants is one of the most important measures of PMTCT prevention of vertical transmission of HIV and EID program effectiveness, which not only acknowledges the availability, and effectiveness of therapeutic interventions but also the competing threats to infant HIV-free survival [3]. Survivorship

in this study was defined in terms of infant HIV status at the end of 12 months whereby the infant is HIV-free between 6 weeks (when the first Polymerase Chain Reaction-PCR test is undertaken) and 12 months of age.

Methods

The study adopted a prospective cohort study design approach whereby mother-infant pairs were followed up for 12 months. The study was conducted in the following public healthcare facilities: Mathare North Health Centre, Mbagathi County Hospital, and Kibera South Health Centre in Nairobi County within the catchment area of Kibra and Mathare informal settlements. These study sites were chosen because approximately 60-70% of urban dwellers in Kenya are believed to be living in the slum area [4, 5] with a 12% prevalence of HIV compared to a 5% prevalence among non-slum dwellers. The study population was infants born to mothers living with HIV enrolled for EID services at Mathare North Health Center, Mbagathi County Hospital, and Kibera South Health Center in Nairobi County.

A total of 166 participants were selected using simple random sampling with the sampling frame being the post-natal registers in the three identified healthcare facilities. The formula for calculating longitudinal studies was used as shown below:

n = [□∝√1+□1□∗ (1−□∗)+□□√□1 (1−□1)/□+□2(1−□2)]2

(□1−□22)

Where

Formatted: Justified, Space After: 0 pt, Line spacing:

P_1 = 0.45 (Proportion of HIV positive infants born to mothers on ART in 2015- GOK, 2016)

P_2 = 0.14 (Proportion of HIV positive infants born to mothers not on ART in 2015 - GOK, 2016)

p1 = response proportion in group 1 (q1 = 1 − p1)

p2 = response proportion in group 2 (q2 = 1 − p2)

$\bar{p}$ = (p1 + p2)/2

$\bar{q}$ = 1 − $\bar{p}$ = 1.96

The study was powered at 0.80 with the level of significance set at 0.05%. A sample size of 166 respondents (after adjusting for 40% loss to follow-up) was selected, using the Cochran formula, with 163 (98.2%) respondents participating in the study. The distribution of infants selected in the three facilities was based on the number of mothers living with HIV that attended the prevention of vertical transmission clinics PMTCT clinics in the selected healthcare facilities between October 2015 and October 2016. They were then selected proportionately based on the number of mothers living with HIV that attended the prevention of vertical transmission clinicsPMTCT clinics in each of these three healthcare facilities. In Mathare North health center (MNHC) 80 infants were selected, in Kibera South health center (KSHC) KSHC 60 infants were selected, while in Mbagathi County hospital (MDH) 26 infants were selected. This gave a total of 166 infants selected to participate in the study. The study was powered at 0.80 with the level of significance set at 0.05%.

Simple random sampling technique was used to select the study participants using tables of random numbers. The postnatal register was used as a sampling frame from which

participants who met the inclusion criteria were selected. Once selected from the postnatal register, the mother-infant pairs were interviewed as they came for EID services from 2 weeks to 6 weeks after birth. The inclusion criteria constituted: HIV-exposed infants at the age of 2 weeks post-delivery up to ≤ 6 weeks of age, HIV-exposed infants whose mothers were registered in the Comprehensive Care Clinic (CCC) of the selected facilities (CCC is where all clients living with HIV seek care and treatment), and HIV-exposed infants whose mothers agreed to participate in the study. The exclusion criteria were HIV-exposed infants whose mothers had visited the CCCs in the selected hospitals but were in transit and, HIV-exposed infants whose mothers were newly transferred from other healthcare facilities (had been in the CCC for less than one year prior to the study onset). These mother-infant pairs were excluded from the study as there were no records to corroborate the history obtained from the mothers.

Recruitment of infants into the study was conducted 2 weeks after delivery. The infants were followed up for 12 months to assess their HIV status. Infants follow up was done in their first year of life as morbidity and mortality among HIV exposed infants is highest within this period. Infant follow-up was scheduled to coincide with the Kenya Expanded Program for Immunization (KEPI) timetable at 6, 10, 14 weeks, and 12 months. The infants were also followed up at 6 months as per the Government of Kenya (GOK) EID schedule (GOK, 2016). Results for the HIV virological testing and other health parameters were collected from HIV-exposed infants at 6 weeks, 6 months, and 12 months. The PCR results were obtained from the PCR testing laboratories as documented in the EID registers of the respective healthcare facilities. A scheduled visit was

considered a delayed visit if the mother failed to return within one week of the scheduled appointment. The EID process from birth to the announcement of the infants' results to the mothers was considered to be ‘‘complete’’ if the PCR tests were performed at the scheduled time points and the final PCR results were provided to mothers at 12 months of the infant's age. The vital status of infants who did not return for a follow-up visit was determined by phone calls at 12 months, and an oral interview was conducted to establish reasons for loss to follow up (LTFU).

A semi-structured interviewer-administered questionnaire was adopted and customized from the World Health Organization data collection and analysis tools for HIV. The semi-structured interviewer-administered questionnaire was used to collect information from the mothers living with HIV by the researcher. The researcher was assisted by six research assistants. Data collection was done at scheduled time points at 6, 10, and 14 weeks, 6 months, and 12 months in the selected healthcare facilities. The semi-structured interviewer-administered questionnaires collected the following data: socio-demographic and socio-economic data of the mother, disclosure of HIV status, maternal characteristics during while attending the prevention of vertical transmission sessions, infant feeding practices, and ART adherence. A data abstraction tool was used to collect quantitative data from the mother’s health records. The data abstraction tool collected information from the mothers' CCC follow-up file on maternal body mass index (BMI), viral load count, HIV staging, ART adherence, ART regimen, and presence of opportunistic and co-infections in the mother. The collected data was presented in tables and charts. To test the validity of the instrument, a pretest of the data collection tools was done at Langata

Health Centre, which has a system almost similar to the selected hospitals and serves the Kibera and Mathare catchment population. In total, 10% (16) of the questionnaires were used to assess the reliability of the questionnaires. The data that was collected was analyzed, summarized and the findings disseminated to the providers at Langata Health Centre. The reliability of the data collection tool was tested using Cronbach's alpha technique whereby reliability indexes obtained in the study were all above 0.7 and deemed acceptable.

Data cleaning, coding, and analysis were done using STATA Version 14. Descriptive statistics were used to explore and summarize the data. ~~Categorical variables were analyzed using measures of central tendency (mean and median), proportions, and frequencies.~~ The HIV-free survival function (the outcome of the study) was assessed using Kaplan- Meier (gave a summary of the HIV-free Survival function and Hazard functions) and Mantel Log-rank was used to test for association between the identified variables. Cox-proportional Hazard test at the multi-variable level tested for the relative risk of the event occurring (infants converting from HIV negative to HIV positive). The Z values and corresponding p-values were reported as well as the Hazard ratios and their corresponding confidence intervals. Cumulative incidence function was used to model for possible competing interests i.e. death and transfer outs. Finally, the proportional hazard assumption using Schoenfield's residual was used to assess the best-fit model by ensuring that the Cox-hazard assumption was met. The level of significance was set at P <0.05 at 95% confidence interval. The study was powered at 80%. All significant variables at p= <0.1 were used in the forward and backward modeling process to determine the best

model using only two prognostic variables. Modeling to choose the most appropriate model from several other competing models was done using Akaike's Information Criteria (AIC). The Schoenfeld residual test was used to assess if the Cox-hazard proportion assumption was met for each model created.

The researcher received ethical approval to conduct the study from the Kenya Medical Research Institute (KEMRI) Scientific Ethics Review Unit (SERU), KEMRI/SERU/CPHR/002/3525. Clearance to carry out the study was given by the Board of Postgraduate Studies (BPS) of Jomo Kenyatta University of Agriculture, Science, and Technology (JKUAT). Permission to carry out the study was also granted by the Nairobi County Ministry of Health and the Medical Superintendent of the facilities where the study was conducted. Informed consent was obtained from the mothers, and the benefits and risks of the study were explained to them too. Confidentiality, privacy, and anonymity were maintained, and participants were free to withdraw from the study without implications for the mother-infant pairs.

Results

All HIV exposed infants recruited into the study were screened for HIV using the PCR test. A total of 166 mother-infant pairs were recruited for the study and 163 (98.2%) mother-infant pairs participated at the beginning of the study (6 weeks). The 3 (1.8%) mother-infant pairs were censored from the study before 6 weeks of age as follows: at two and a half weeks one infant transferred out of Mathare North Health Center, at three

weeks one infant died and at five weeks another one infant died. At six months, 22 mother-infant pairs were LTFU, and 10 more mothers were LTFU at 12 months.

The median age of the mothers was 29 years (IQR: 25-33)Most 75(45%) of the mothers were aged between 25-34 years while the rest were aged between 18-24 years and 35-44 years, 34(20%) and 57 (34.3%) at recruitment. Slightly above half 93 (56.0%) of themthe participants had a 93 (56.0%) having a primary level of education or below. The majority of the mothers were married 141 (84.9%) at recruitment and 113 (86.3%) at the endpoint. Most of the mothers had informal employment 108 (65.1%) at recruitment and 79 (60.3%) at endpoint, with slightly above half of the mothers 95 (57.2%) with a household income that ranged between Ksh. 6001-12000 (Table 1).

Table 1. Socio-demographic and socio-economic characteristics of HIV positive mothers

**Maternal characteristics**	**Time-points**	
	**Recruitment (6 weeks)**	**Endpoint (12 months)**
**n=166**	**Frequency (%) (n=163)**	**Frequency (%) (n=131)**
**Age in completed years**		
18-24	34(20.5)	27(20.6)
25-34	75(45.2)	58(44.3)
35-44	57(34.3)	46(35.1)
**Highest Level of Education**		
≤Primary	93(56.0)	75(57.3)
≥Secondary	73(44.0)	56(42.8)
**No. of persons living in the household**		
2-5	121(72.9)	88(67.2)
6-8	45(27.1)	43(32.8)
**Respondent marital status**		
Single	25(15.1)	18(12.7)
Married	141(84.9)	113(86.3)

**Employment status**		
Formal	27(16.3)	21(16.0)
Informal	108(65.1)	79(60.3)
Unemployed	31(18.6)	31(23.7)
**Monthly income (Ksh.)**		
≤6000	34(20.5)	37(28.2)
6001-12000	95(57.2)	67(51.2)
≥ 12001-18000	37(22.3)	27(20.6)

The incidence rate was 9 cases per 100 person-years (CI 5.43, 6.10) was obtained at the end of the 12-month study period. The failure event was defined as an infant with a positive PCR test during the study period with total failures being 13 (9.41%) at the end of the study time point (12 months). Slightly above half 85 (52.15%) of the infants had timely recruitment (≤ 6 weeks) for EID service delivery.

Kaplan-Meier HIV-free survival estimate curve over a one-year follow-up

Kaplan–Meier estimate curves showed that the probability HIV-free survival of an infant over a 12-month follow-up period. (Fig 1).

Fig 1: Kaplan-Meier infant HIV-free survival estimate over a one-year follow-up period.

Kaplan-Meier infant failure estimate curve over a one-year follow-up

Kaplan-Meier failure estimate curves showed that the probability of HIV vertical transmission of an infant over a 12-month follow-up period was 0.09350 cases per person-year which translated to 9 cases per 100 person-years (95% CI 5.43, 16.10). Thirteen infants turned HIV positive over the 12-month follow-up period (Fig 2).

Fig 2: Kaplan-Meier infant hazard failure estimate over a one-year follow-up period

Mantel log-rank HIV-free survival estimates for HIV-exposed infants over a one-year follow-up period

The Mantel log-rank test was used to determine factors associated with HIV-free survival at univariate level. This is as shown in (Table 2). At recruitment (6weeks) 161 (97.0%) mothers reported that they were exclusively breastfeeding their infants while 3 (1.8%) did mixed feeding and 2 (1.2%) were on formula milk. Only 75 (53.2%) of the infants were exclusively breastfed for six months, while 64 (45.4%) were mixed fed and 2 (1.4%) were on formula milk for the first six months of their lives. Cross tabulations for infant feeding practices were not undertaken as there was no way to corroborate the mothers report on the infant feeding practices during the study period. All the infants were put on prophylaxis from birth up to 6 weeks where their HIV status was determined via a PCR test for further care and treatment support. At 6months and 12 months, PCR tests were repeated on the infants to determine those that needed to be switched on ART, following a positive HIV result.

Table 2. Maternal factors associated with infant HIV-free survival.

Prognostic factors (n = 131)	Chi-square test	p-value	DF
**Maternal Socio-demographic and economic characteristics**			
Age in completed years	7.39	**0.025**	2
Highest Level of Education	0.12	0.733	1
No. of persons living in the household	0.04	0.841	1
Respondent marital status	3.41	0.065	1
Employment status	23.93	**<0.001**	2
Household Monthly income (Ksh)	17.98	**0.001**	2
**Maternal characteristics during PMTCT clinic visits**			
Year Confirmed Positive	11.86	**<0.001**	1
Mode of Delivery	0.18	0.668	1
Given ART in pregnancy	12.66	**<0.001**	1
Stage ART administered in pregnancy	20.66	**<0.001**	2
Gestation of pregnancy at 1st PMTCT visit	0.16	0.923	2
No of PMTCT clinic vVisits	19.58	**<0.001**	2
**Maternal disclosure status**			
Have a partner	0.24	0.627	1
Disclosed status to the partner	9.64	**0.002**	1
Know Partner's HIV status	10.80	**0.001**	1
Spouse HIV Status	0.17	0.678	1
**Maternal characteristics at 12 Months**			
ART adherence (Morisky Score)	33.71	**<0.001**	2
Maternal HIV Staging	26.94	**<0.001**	1
ART Regimen	81.58	**<0.001**	1
Viral Load (VL)	42.24	**<0.001**	2
Maternal BMI	17.23	**0.001**	3
**Timeliness into EID enrollment at 6 weeks (recruitment) among exposed infants**			
Timeliness at 6 weeks (recruitment)	5.22	**0.022**	1

Cox Regression analysis for socio-economic and demographic factors showing HR and AHR over 12 months follow-up period

Table 3 gives a summary of the Cox PH regression analysis for socio-demographic and socio-economic factors, maternal factors during PMTCT,the attendance of prevention of vertical transmission clinics (PMTCT clinics) and the corresponding hazard ratios and adjusted hazard ratios.

Table 3. Cox Regression analysis of socio-economic and demographic factors, showing HR and AHR over 12 months follow-up

Time point (n= 166)	Infant HIV-free survival over the 12-month follow-up period	
**Maternal characteristics**	**HR (95%CI)**	**AHR (95%CI)**
**Age in years**		
35-44 *(Ref)*	1.00	
18-24	**5.97(1.20, 29.58)**	
25-34	1.96 (0.38, 10.13)	
**Employment**		
Unemployed *(Ref)*	1.00	
Formal	0.14 (0.02, 1.10)	
Informal	**0.07 (0.02, 0.31)**	
**Monthly Household Income**		
≤ 6000 *(Ref)*	1.00	
6001-12000	**0.10 (0.02, 0.44)**	
≥12001-18000	**0.12 (0.02, 0.10)**	
**Year confirmed +ve**		
> 2 years since positive HIV diagnosis	1.00	
≤ 2 years since positive HIV diagnosis	**6.97(1.96, 24.76)**	

Cox Regression analysis for disclosure status and ~~PMTCT~~ prevention of vertical transmission factors showing HR and AHR over 12 months follow-up.

Table 4 gives a summary of the Cox ~~-PH~~ regression analysis for maternal disclosure status and maternal factors at 12 months and the corresponding hazard ratios and adjusted hazard ratios.

Table 4. Cox Regression analysis for disclosure status and ~~PMTCT~~prevention of vertical transmission factors showing HR and ARH over 12 months follow-up period

Time point (n= 166)	Infant Survival over 12M follow-up period	
**Maternal characteristics**	**HR (95%CI)**	**AHR (95%CI)**
**Disclosed status to partner**		
Yes *(Ref)*	1.00	
No	**5.10 (1.65, 15.82)**	
**Know partner's HIV status**		
Yes *(Ref)*	1.00	1.00
No	**5.87 (1.80, 19.16)**	**4.56(1.27, 16.45)**
**ART Adherence**		
Poor *(Ref)*	1.00	
Good	**0.05 (0.01, 0.23)**	
Inadequate	**0.14 (0.04, 0.50)**	
**HIV Staging**		
I *(Ref)*	1.00	
II	**17.84 (3.94, 80.89)**	
**Viral Load (VL)**		
High VL *(Ref)*	1.00	
...Undetectable VL	**0.02 (0.01, 0.17)**	
...Low VL	**0.12 (0.02,0.90)**	
**...ART Regimen**		
...Second-line *(Ref)*	1.00	1.00
...First-line	**0.03 (0.01, 0.10)**	**0.03 (0.01, 0.08)**
**...Given ART during pregnancy**		
...No *(Ref)*	1.00	
...Yes	**0.11 (0.02, 0.52)**	
**...Stage ART admin in Pregnancy**		
...Third Trimester *(Ref)*	1.00	
...Frist Trimester	**0.09 (0.02, 0.31)**	
...Second Trimester	0.38 (0.10, 1.45)	
**...No. of PMTCT visits attended**		
...2 *(Ref)*	1.00	
...≤1	0.86 (0.23, 3.26)	
...≥3	**0.12 (0.03, 0.40)**	**0.04 (0.01, 0.25)**
**...Maternal BMI**		
…Normal *(Ref)*	1.00	
...Underweight	**6.29 (1.93, 20.47)**	
...Overweight	0.64 (0.12, 3.26)	
...Obese	0.80 (0.09, 6.90)	
**...Timeliness into EID enrollment**		
...Timely (≤6weeks) *(Ref)*	1.00	
...Delayed (>6weeks)	**4.00 (1.12, 14.32)**	

Final model of maternal factors influencing infant HIV-free survival

All significant variables at p= <0.1 were included in the modelling process to determine the best model using only two prognostic variables. ~~Infant death was tested to find out if it was a competing risk factor but was found to be insignificant (i.e. was not a competing risk factor).~~ Cumulative incidence function was used to model for possible competing interests i.e. death and transfer outs. In the final HIV-free survival model, mothers on first-line ART regimen and had attended more than three PMTCT clinic visits and, knew their partner's HIV status had lower hazard ratios of experiencing infant HIV vertical transmission, relative to mothers that were on the second-line ART regimen, had attended fewer antenatal clinic (ANC) visits and did not know their partners HIV status respectively (Table 5).

Table 5. Final model of maternal factors influencing HIV-free survival of infants

Maternal characteristics	Hazard Ratio	Robust Std. Err.	z	P>z	[95%C. I] Lower	Upper	Prob > Chi2
							**<0.001**
**ART Regimen**							
Second-line *(Ref)*	1.000						
First-line	0.028	0.016	-6.3	**<0.001**	0.0091	0.083	
**No. of PMTCT clinic visits**							
2 *(Ref)*	1.000						
≤1	0.536	0.485	-0.6	0.491	0.091	3.162	
≥3	0.046	0.040	-3.5	**<0.001**	0.008	0.255	
**Know partner's HIV status**							
Yes *(Ref)*	1.000						
No	4.564	2.9853	2.32	**0.020**	1.266	16.448	
**Summary of final HIV-free survival model at 12 months**							
**Model p-value**	**n**	**Log-Likelihood**	**AIC**	**BIC**	**Schofield residual test**		**DF**

<0.001	131	-30.15	68.32	79.62	0.86	4

Discussion

Infant HIV-free survival in HIV-exposed infants is one of the most important measures of PMTCT and EID programs effectiveness, which not only acknowledges the availability, and effectiveness of therapeutic interventions but also the competing threats to infant HIV-free survival [3]. In the current study, HIV-free survival among the HIV-exposed infants was estimated at 90.7% (with a total person-time of 139.02 days, 95% CI 5.43, 16.10) for the infants aged between 6 weeks and 12 months a finding similar to that in Rwanda 91.9% [6] but lower than what was observed in Zambia 96.3% (95% CI: 94.8, 97.4%) [7], and much higher than in Cameroon (72.6%; 95% CI: 62.3, 80.5) and South Africa (77.7%; 95% CI: 72.5, 82.1) [8].

In a similar study longitudinal study undertaken in Malawi [9] the MTCT vertical transmission rate of HIV rate was 4.9% (95% CI 3.7, 6.4%), much lower than what was observed in the current study i.e., 9.41%. The authors attributed poor infant HIV-free survival to factors similar as those seen in the current study i.e. mothers not disclosing their HIV status to their partners, poor maternal ART adherence and late maternal ART initiation. (whereby, mothers were initiated on ART during pregnancy or during the post-partum period). South Africa [10] had a similar MTCT vertical transmission rate of HIV rate as that seen in Malawi [9] but much lower in than in the current study. However, reasons for poor infant HIV-free survival in the South African study [10] differed from what was seen in Malawi and Kenya, and was attributed to unknown maternal CD4-cell-

count ~~(adjusted HR (aHR 2.66 (95% CI 1.5, 5.6),~~ undocumented maternal HIV status ~~(aHR 2.21 (95% CI 1.0, 4.7)~~ and exclusive ~~(aHR 2.3 (95% CI 1.0, 5.2)~~ or mixed breastfeeding ~~(aHR 3.7 (95% CI 1.2, 11.4)~~ [10]. Maternal age influenced infant HIV-free survival over the 12 months follow-up period whereby younger mothers aged between 18-24 years had a higher hazard ratio (5.97) of infant HIV vertical transmission relative to their older counterparts 36-47 years, a finding similar to what was observed in Rwanda, Malawi, ~~and~~ Swaziland, and Kinshasa [7, 9, 11, 12] respectively whereby young maternal age was associated with an increased risk of infant HIV vertical transmission. This however differed from findings observed in South Africa whereby young maternal age was not associated with infant HIV- free survival [13~~2~~]. ~~On the contrary, a study in Kinshasa [13] demonstrated that younger maternal age was associated with positive child outcomes in terms of reduced child morbidity, mortality, and better EID uptake.~~

Unemployment and low household incomes were facilitators of poor infant HIV-free survival in the current study. Poverty increases the chances of infant HIV vertical transmission [13]. Diagnosing mothers early before pregnancy and putting them on ART improves infant HIV-free survival and reduces infant chances of HIV infection. This was supported by [14] where the authors found that failure to diagnose HIV infection during early pregnancy was a primary reason for missing both prevention of vertical transmission of HIV (PMTCT) and EID interventions contributing disproportionately to new pediatric infections.

~~Mothers who had some form of employment and had higher monthly household income levels had a lower hazard ratio (HR= 0.07) of infant HIV vertical transmission, relative to~~

Formatted: Highlight

those who were unemployed and had a low monthly household income of Ksh. <6000. Poverty is a facilitator of poor infant HIV free survival and interventions to economically empower mothers living with HIV would improve their health and subsequent infant HIV free survival [13].

Diagnosing mothers early before pregnancy and putting them on ART improves infant HIV free survival and reduces infant chances of HIV infection. This was supported by [14] where the authors found that failure to diagnose HIV infection during early pregnancy was a primary reason for missing both PMTCT and EID interventions contributing disproportionately to pediatric new infections. In the current study, mothers who had a recent HIV positive diagnosis (within two years of HIV diagnosis) had a higher hazard ratio (HR= 6.97) of infant HIV vertical transmission relative to mothers that had lived with HIV for a period of more than two years. Diagnosis during pregnancy poses a challenge for disclosure and sometimes drug compliance and consequently increases the risk of infant HIV vertical transmission [15].

Mothers who received ART during pregnancy had a lower hazard (HR=0.11) of infant HIV vertical transmission relative to mothers that had not received ART during pregnancy a finding similar to a Rwandan and Ethiopian studiesdy undertaken at a national level [6, 16] respectively. Further, a study in Ethiopia [16] adds to say that detection of maternal HIV status in early pregnancy through voluntary counseling and testing is a key factor in ensuring infant HIV free survival because then ART can be started as early as possible reducing the chances of vertical transmission.

Attending more than three PMTCT clinic visits had a lower hazard ratio (HR= 0.12) of infant HIV vertical transmission relative to mothers who had attended lesser PMTCT clinic visits., for instance, only two visits. In a retrospective study conducted in Zambia [17] mothers that received antenatal care yielded an estimated 89.8% (95% CI: 86.8, 92.2) infant HIV-free survival, and a lower MTCT vertical transmission of HIV rate and higher retention rates to the PMTCT clinics. The authors also found that early identification of mothers living with HIV and starting PMTCT visits within the first trimester of pregnancy were associated with higher retention rates in the PMTCT clinics. A study in Lodwar, Kenya [18] found that mothers who attended more than one PMTCT clinic had higher odds of 2.78 (CI: 1.25, 6.17) of administering prophylaxis to their infants relative to mothers who had attended less than one PMTCT clinic visit. This was found to reduce MTCT of HIV and infant morbidity.

In the current study, non-disclosure of HIV status and not knowing a partner's HIV statusmothers who failed to disclose their HIV status to their partners nor knew their partner's HIV status experienced ahad higher hazard ratio (HR= 5.10) and (HR= 5.87) respectively of infant HIV vertical transmission relative to mothers that had disclosed their status and knew their partners HIV status. Male partner involvement through participation in ANC attendance has been associated with enhanced uptake of PMTCT prevention of vertical transmission of HIV services specifically, improvements in HIV testing and partner disclosure, ART adherence, and improved infant feeding strategies [2]. Lack of male partner participation resulted in reduced infant HIV-free survival (HR= 3.95, 95% CI: 1.21, 12.89; p=0.023), which was maintained when adjusting for maternal

Formatted: Highlight

Formatted: Highlight

and infant ARV use (AHR=3.79, 95% CI: 1.15, 12.42) [2]. A study conducted in Papua New Guinea revealed lower infant HIV-free survival (in terms of infant death and infant HIV vertical transmission) and loss to follow-up in mothers that had reported untested or unknown partner HIV status [19]. However, in Malawi, the findings revealed that male partner involvement was not associated with lower risk of infant HIV vertical transmission [20]. In South Africa male partners were .found to provide access to medical services whenever the mothers needed them to do so [21]

Non-disclosure of maternal HIV status to their partners and lack of knowledge of their partner's HIV status in the current study had higher hazards of infant HIV vertical transmission, a finding corroborated by other authors whereby male partners who had disclosed their HIV status to the mothers had a greater likelihood of PMTCT involvement and were more involved with the infant [2]. Male partners were also found to provide access to medical services whenever the mothers needed them to do so. Fear of stigma relating to maternal HIV status was associated with high levels of non-disclosure that affected ART adherence and PMTCT attendance, factors that negatively affected infant HIV-free survival [21]. The authors recommended community education to reduce stigma and discrimination and male partner involvement during PMTCT to enhance HIV disclosure. Male participation during PMTCT clinic attendance improved maternal sexual and reproductive health, reduced stigma and discrimination, and consequently improved infant health outcomes [22]. Non-disclosure of maternal HIV status was associated with an increased risk of vertical transmission of HIV to infants and high maternal viral load [23]. The authors further stated thaAdditionally t male involvement increased the

Formatted: Highlight

Formatted: Highlight

Formatted: Highlight

incidence of HIV testing and counseling and facilitated partner disclosure [24]. ~~Non-disclosure of maternal HIV status was associated with an increased risk of vertical transmission of HIV to infants and high maternal viral load [23].~~

~~Fear of stigma and discrimination has been demonstrated to lead to non-disclosure of maternal HIV status to their significant others, affecting ART adherence and viral load counts [18]. This then affects infant HIV free survival, increasing the risk of vertical transmission during pregnancy and the breastfeeding period. A few mothers, however, reported tremendous partner support following the disclosure of their HIV status in the current study.~~

~~Partner involvement and support through adherence counseling and other PMTCT interventions improve overall maternal adherence and retention in the EID clinic ultimately improving infant HIV free survival [24].~~ Women in sub-Saharan Africa reported that they faced difficulty in negotiating the use of condoms with their male partners despite the fact that they were aware that some of their partners had multiple sexual partners [25]. A few mothers reported that their partners refused to take their ART, and some even refused to go for HIV testing. ~~All these n~~Negative male partner behaviors put both the mothers and infants at increased risk of high viral loads and vertical transmission~~, respectively~~ [26].

~~In the current study, mothers with more advanced disease of HIV infection, for instance, those in Stage 2 had a higher hazard ratio of experiencing infant HIV vertical transmission relative to mothers that were classified as being in stage 1 of HIV infection (as per the World Health Organization HIV classification). These findings were similar to~~

Formatted: Highlight

Formatted: Highlight

Formatted: Highlight

Formatted: Highlight

those of a study undertaken in Johannesburg [14] where mothers with advanced disease experienced lower infant HIV-free survival compared to mothers with less advanced disease who had CD4 counts ≥ 350 cells/ml. Mothers with undetectable and low viral loads had a lower hazard ratio of infant HIV vertical transmission relative to mothers who presented with high viral load levels in the current study. These findings were similar to those of [17] whereby mothers with advanced disease had increased mortality and morbidity in their infants. However, in a South African study, [14] maternal viral load was only significantly associated with infant HIV-free survival at the bi-variable level and was no longer a predictive factor of infant HIV-free survival at the multi-variable level.

Mothers on the first-line ART regimen in the current study experienced a lower hazard of infant HIV vertical transmission relative to mothers on the second-line regimen. PMTCT regimens act to lower maternal and infant viral load limiting the establishment of the infant viral reservoir and lowering the maternal viral load and HIV progression. Therefore, in the periods when the mothers living with HIV are being switched from the first to second-line regimens there are usually periods of high maternal viremia that could increase the risk of vertical transmission to the infant [14]. However, other non-biological explanations also play a role in infant HIV vertical transmission. Mothers who were changed from one regimen to another during pregnancy or breastfeeding for any reason had high odds of infant HIV vertical transmission and their infants were also virally unsuppressed (AOR 1.82 CI: 1.21, 2.27) [26].

Formatted: Highlight

Formatted: Highlight

Underweight mothers had a higher hazard ratio (HR= 6.29) of infant HIV vertical transmission relative to mothers with a normal BMI. A study in Rural Kenya demonstrated that poor nutritional status in mothers living with HIV may impair immunity by weakening the epithelial integrity of the body cells, which in turn increases the risk of infant HIV vertical transmission [27].

Close to a half 81 (48.0%) of the infants had late initiation into the EID (> 6 weeks of age) which led to a higher hazard ratio (4.00) of infant HIV vertical transmission relative to mothers who had enrolled their infants for EID at 6 weeks or earlier. ~~According to~~ T~~t~~he GOK 2016 guidelines [3] recommend all HIV exposed infants should be enrolled for EID any time from birth up to 6 weeks of age. ~~Any enrollment (> 6 weeks) is therefore classified as a delayed enrollment and has been associated with poor infant health outcomes and increased risk of loss to follow up.~~ Globally, it is estimated that nearly half of HIV-exposed infants receive an EID test within the first two months but with wide variations in initiation timeliness in various countries [28]. The success of infant HIV ~~testing~~ programs is highly dependent on the early identification and enrollment of mother-infant pairs~~s~~ in care and/or treatment [29].~~to the PMTCT services and systematic follow-up until the final determination of the infant's HIV status upon cessation of breastfeeding [29].~~ A study conducted in four countries in Asia and Africa (Cambodia, Senegal, Namibia, and Uganda) elicited that the median age of testing among infants referred from PMTCT ~~services~~clinics was approximately 2 months over the life of the program, with less than 50% of these infants being tested well after 2 months of age [30].

A retrospective study in Tamil found that the median age at initiation into EID at the time of sampling was 4 months [31]. Only 13% of the infants living with HIV were tested within 6 weeks of birth, 29% had their first test by 4 months, 52% by 6 months, and 85% by 12 months. Although guidelines recommend testing exposed children at 6 weeks of age, the study found that less than one-fifth of eligible infants were tested then. Long turn-around times between the first PCR test and the second confirmatory PCR test resulted in high attrition for the mother-infant pair, which caused inappropriate delays in starting children living with HIV on life-saving ART. The authors recommended that a dried blood sample (DBS) positive result should be used to start the infants on ART immediately as opposed to delaying treatment as a second confirmatory full blood test is awaited to capitalize on the overwhelming benefits of early ART initiation for infants and reduce the numbers of LTFU.

In Rural Kenya, [32] found out that out of the 233 infants enrolled in the EID program only 75 (32%) were enrolled for care within the first two months of life. The median age for enrollment was 5 months. Moreover 60 (43%) of the 139 dropouts occurred within 2 months after enrolment with only 74 (32%) of the infants retained up to 18 months. Factors that attributed to late initiation, LTFU, and dropout included younger mothers, inadequate caregiver knowledge on EID processes and interpretation of the same, lack of appropriate social support structures to facilitate disclosure and handle stigma, indirect costs as one travels for the test results, and drugs in the healthcare facility. Other factors included denial by the caregiver especially if the infant is found to have a positive

Formatted: Highlight

Formatted: Highlight

virological test, concerns about the pricking during testing being painful for the infant and poor interpersonal relationships with the healthcare providers.

This study had several limitations. Firstly, exposure history was from the mother's self-report, and not all the history that was given could be corroborated from the maternal CCC file, Integrated Management of Childhood Illness (IMCI) booklet, and appointment booklets. Where it was not possible to corroborate the mother's self-report, the questionnaires were structured to counter check the responses asked in a different format within the same questionnaire. Secondly, given the phrasing of the questionnaire, it was difficult to determine if the mothers were adherent to ART throughout the pregnancy period, for instance if there were periods of cessation and non-adherence. Adherence, however, was also determined by observation of participant serial viral loads from pregnancy up to one-year post-partum. Due to the small sample size selected, the incidence rate of 9 cases per 100 person-years (95% CI: 5.465 – 16.290) may have been an underestimation of the true MTCT vertical transmission of HIV rates. The small sample size gave wide confidence intervals (CIs) (5.5-16.3) which is not as useful for health policy guidance as narrow CI's. Another limitation of our study was the lack of detailed data regarding clients that were lost to follow-up since it was not possible to reach them on the phone. Lastly, another limitation of the study was that data on feeding practices were provided in the manuscript. However bivariable and multivariable analysis on the same was not done because there was no way to corroborate the breastfeeding and other feeding options from the mother's narrative, at the time of the study. A great strength of the study was in the design of the study, whereby a longitudinal study design

was applied, and participant outcomes were studied over a period of 12 months using ~~that~~ survival analysis.

Conclusion

Scaling up care and treatment for mother-infant pairs more so in resource-limited settings improves overall HIV-free survival among HIV-exposed infants. There is a need to create a special package of care for the younger mothers aged (18-24) and mothers with recent HIV diagnoses (i.e., less than 2 years since HIV positive diagnosis). The special package should have more rigorous adherence and nutritional counseling, with close monitoring of these mother-infant pairs. This should also be accompanied by robust partner HIV status disclosure strategies to enhance partner support for mothers attending the EID services of HIV. These strategies are aimed at improving infant retention and HIV-free survival throughout the EID cascade of care.

Acknowledgements

I wish to express my most heartfelt and sincere gratitude to the almighty God for the good health, protection, and care that He has granted unto me. My sincere gratitude goes out to all the healthcare workers, healthcare facility in-charges, and the study participants for their support and cooperation, and to all those who assisted in any way during the data collection and analysis period. Special appreciation goes to Prof. Thomas Achia Center for Disease Control (CDC), KEMRI who played a great role in statistical mentorship.

Author contributions

Conceptualization: Elizabeth M. Kiilu

Data curation: Prof. Achia, Elizabeth M. Kiilu

Formal analysis: Prof. Achia, Elizabeth M. Kiilu, Samuel Mutua, Mary Kamina

Investigation: Elizabeth M. Kiilu

Methodology: Elizabeth M. Kiilu, Simon Karanja, Gideon Kikuvi

Project administration: Elizabeth M. Kiilu

Supervision: Simon Karanja, Gideon Kikuvi, Peter Wanzala

Writing- Original draft: Elizabeth M. Kiilu

Writing- Review & editing: Elizabeth M. Kiilu, Simon Karanja, Gideon Kikuvi, Peter Wanzala

References

1. World Health Organization. Validation of elimination of mother-to-child transmission of HIV, syphilis, and hepatitis B. Geneva. 2023.
2. Alusio A, Bosire R, Gatuguta A, Kiarie J, Nduati R, John-Stewart G, et al. Male Partner Participation in Antenatal Clinic Services is Associated with Improved HIV-free survival Among Infants in Nairobi, Kenya: A Prospective Cohort Study. Journal of Acquired Immune Deficiency Syndrome. 2016;169-174.

3. Government of Kenya. Kenya Framework for the Elimination of Mother-To-Child Transmission of HIV and Syphilis. 2016.
4. UN-Habitat. The state of African Cities 2014: Re-imagining sustainable urban transitions. 2014.
5. Corburn J, Hildebrand C. Slum Sanitation and the Social Determinants of Women's Health in Nairobi, Kenya. Journal of Environmental and Public Health. 2015; 2015:6.
6. Ruton H, Mugwaneza P, Shema N, Lyambabaje A, Bizimana JD, Tsague L, et al. HIV-free survival among nine- to 24-month-old children born to HIV-positive mothers in the Rwandan national PMTCT program: A community-based household survey. Journal of the International AIDS Society. 2012;15(4).
7. Chi BH, Mutale W, Winston J, Phiri W, Price JT, Mwiche A, et al. Infant Human Immunodeficiency Virus-free Survival in the Era of Universal Antiretroviral Therapy for Pregnant and Breastfeeding Women: A Community-based Cohort Study from Rural Zambia. Pediatric Infectious Diseases Journal. 2018; 37(11):1137-1141.
8. Stringer JSA, Stinson K, Tih P, Giganti M, Ekouevi DK, Creek T. et al. Measuring Coverage in MNCH: Population HIV-Free Survival among Children under Two Years of Age in Four African Countries. PLoS Medicine. 2013;10(5).
9. Lettow MV, Barr BA, Oosterhout JJ, Schouten E, Jahn A, Kalua T, et al. The National Evaluation of Malawi's PMTCT Program (NEMAPP) study: 24-month HIV-exposed infant outcomes from a prospective cohort study. HIV Medicine. 2021;23:573-584.

10. Goga AE, Lombard C, Jackson D, Ramokolo V, Ngandu NK, Sherman G, et al. Impact of breastfeeding, maternal antiretroviral treatment, and health service factors on 18-month vertical transmission of HIV and HIV-free survival: results from a nationally representative HIV-exposed infant cohort, South Africa. Journal of Epidemiology & Community Health 2020;74:1069-1077.

11. Chouraya C, Machekano R, Mthethwa S, Lindan K, Mirira M, Kudiabor K, et al. Mother-to-Child Transmission of HIV and HIV-Free Survival in Swaziland: A Community-Based Household Survey. AIDS and Behavior. 2018;22:105-113.

~~11.~~12. Ditekemena J, Matendo R, Colebunders R, Koole O, Bielen G, Nkuna, M. et al. Health Outcomes of Infants in a PMTCT Program in Kinshasa. Antiretroviral Therapy. 2015;14(5): 449-454.

~~12.~~13. Sifunda S, Peltzer K, Rodriguez VJ, Mandell LN, Lee TK, Ramlagan S, et al. (2019) Impact of male partner involvement on mother-to-child transmission of HIV and HIV-free survival among HIV-exposed infants in rural South Africa: Results from a two-phase randomized controlled trial. PLoS ONE. 2019;14(6): e0217467.

~~13. Ditekemena J, Matendo R, Colebunders R, Koole O, Bielen G, Nkuna, M. et al. Health Outcomes of Infants in a PMTCT Program in Kinshasa. Antiretroviral Therapy. 2015;14(5): 449-454.~~

14. Abrams EJ, Woldesenbet S, Silva JS, Coovadia A, Black V, Technau KG. et al. Despite Access to Antiretrovirals for Prevention and Treatment High Rates of Mortality Persist Among HIV-infected Infants and Young Children. Pediatric Infectious Diseases Journal. 2017;36(6): 595-601.

15. Agudu NA, Ramadhani HO, Isah C, Erekaha S, Fan-Osuala C, Anaba U. et al. The Impact of Structured Mentor Mother Programs on Presentation for Early Infant Diagnosis Testing in Rural North-Central Nigeria: A Prospective Paired Cohort Study. Journal of Acquired Immune Deficiency Syndrome. 2017;75: S182-S189.
16. Derebe G, Biadgilign S, Trivelli M, Hundessa G, Robi Z, Gebre-Mariam M. et al. Determinant and outcome of early diagnosis of HIV infection among HIV-exposed infants in southwest Ethiopia. BMC. 2014;7(309).
17. Buzdugan, R., McCoy, S., Watadzaushe, C., Kang, D. M., Petersen, M., Dirawo, J., et al. Evaluating the Impact of Zimbabwe's Prevention of Mother-to-Child HIV Transmission Program: Mother-to-Child HIV Transmission Program: Mother-to-Child HIV Transmission Program: Population-Level Estimates of HIV-Free Infant Survival Pre-Option A. PLoS ONE. 2015;10(8):e0134571
18. Ongaki D, Obonyo M, James R. Factors Affecting Uptake of PMTCT Services, Lodwar County Referral Hospital, Turkana County, Kenya. Association of Providers of AIDS Care. 2019;1(4):1-4.
19. Carmone A, Bomai K, Bongi W, Frank T, Dalepa H, Lofia B, et al. Partner testing, linkage to care, and HIV Free Survival in a Program to Prevent Mother to Child Transmission of HIV in the highlands of Pupa New Guinea. Global Health Action. 2014;(27)7.
20. Kalembo F, Zgambo M, Mulanga A, Yukai D, Ahmanrd, N. Association between male partner involvement and the uptake of PMTCT interventions in Mwanza District, Malawi: A retrospective cohort study. PLoS One. 2013; 8(6): e66517.

21. Adeniyi Vo, Thomson E, Goon D, Ajayi I. Disclosure, stigma if HIV positive child and access to Early Infant Diagnosis in the rural communities of Tambo District, South Africa: A qualitative exploration of maternal perspective. BMC Pediatrics. 2015;15(98).

22. Yah CS, Tambo E. Why is mother-to-child transmission (MTCT) of HIV a continual threat to newborns in sub-Saharan Africa (sSA). Journal of Infection and Public Health. 2019;12(2019):213-222.

23. Tshweneagae G, Oss V, Mgutshini, T. Disclosure of HIV status to sexual partners by people living with HIV. Curationis. 2015;38(1): E1-6.

24. Imbaya C, Odhiambo-Otieno G, Okello AB. Socio-demographic characteristics, Antenatal Clinic attendance, and PMTCT knowledge of postnatal HIV+ women at an urban public health facility in Nairobi. East and Central Africa Medical Journal. 2014; 1(1):33-40.

25. Thomson K, Telfer B, Opondo AP, Munge J, Ngunga M, Reid, A. Navigating the risks of prevention of mother o child transmission (PMTCT) of HIV services in Kibera, Kenya: Barriers to engaging and remaining in care. PLoS ONE. 2018;13 (1): e0191463

26. Humphrey J, Genberg B, Keter A, Musick B, Apondi E, Gardner A, et al. Viral suppression among children and their caregivers living with HIV in western Kenya. Journal of International AIDS Society. 2019;22: e25272.

26. Matseke MG, Ruiter RAC, Rodriguez VJ, Peltzer K, Setswe G, et al. Factors Associated with Male Partner Involvement in Programs for the Prevention of

Mother-to-Child Transmission of HIV in Rural South Africa. International Journal of Environmental Research and Public Health. 2017; 14(11):1333.

27. Nabwera H.M, Jepkosgei J, Muraya KW, Hassan AS, Molyneux CS, Ali R, et al. What influences feeding decisions for HIV-exposed infants in rural Kenya? International Breastfeeding Journal. 2017;12(31).

28. Essajee S, Bhairavabhotla R, Penazzato M, Kiragu K, Jani I, Carmona S, et al. Scale-up of Early Infant HIV Diagnosis and Improving Access to Pediatric HIV Care in Global Plan Countries: Past and Future Perspectives. Journal of Acquired Immune Deficiency Syndrome. 2017;75(1): S51-S58.

29. Mondi S, Broyles L, Montandon M, Ltoh M, Ochanda B, Langat A, et al. Beyond Early Infant Diagnosis: Changing the Approach to HIV Exposed Infants. Journal of Acquired Immune Deficiency Syndrome. 2018;78(2):S107-S114.

30. Anirban C, Sangeeta T, Robert G, Ndapewa H, Sok P, Charles K, et al. Implementing services for Early Infant Diagnosis (EID) of HIV: a comparative descriptive analysis of national programs in four countries. BioMed Central Public Health. 2011;11:553.

31. Hanna L, Siromany V, Annamalai M, Karunaianantham R, Swaminathan S. Challenges in the early diagnosis of HIV infection in infants: Experience from Tamil Nadu, India. Indian Pediatrics. 2015;52:307-309.

32. Amin HS, Erick SM, Helen NM, Miriam TM, Robert KM, Eduard SJ. Dynamics and Constraints of Early Infant Diagnosis of HIV infection in rural Kenya. AIDS and Behaviour. 2012;16(1):5-12.

Supporting information

S1 Fig. 1 Kaplan-Meier infant survival estimate over a one-year follow-up period.

S2 Fig. 2 Kaplan-Meier infant hazard failure estimate over a one-year follow-up period.

Minimal underlying dataset.

Response to reviewers 24/06/2023	
**Reviewers Comments**	**Changes effected by the PI**
**REVIEWER 2**	
**General comments**	
It is suggested that the discussion section be shortened	This has been shortened from 7 to about 4 pages
No clean (i.e., not tracked) copy was included in the submission. This has made it difficult to read.	A clean version will be provided.
Font size for headings is still inconsistent	All heading font size made consistent.
In the HIV field, there is a move towards using more patient-centered language and avoiding the term mother-to-child-transmission (use 'Vertical Transmission'). This has not been addressed in the Abstract or Manuscript.	In Kenya, the program is still called Prevention of Mother to Child Transmission (PMTCT), I will retain this while making reference to the program/clinic. I have however changed from PMTCT to vertical transmission of HIV while referring to the vertical transmission in the discussions.<br>This is mainly because you cannot call it a prevention of vertical transmission of HIV clinic, it's still called a PMTCT clinic/Program.
Breastfeeding is an important variable when considering vertical transmission of HIV after the first couple of weeks. I don't see reference to breast-feeding	Reference on breastfeeding has been included.
Infant post-exposure prophylaxis is also an important variable in vertical HIV transmission and is not mentioned, not even the guidelines	This has now been reported
**Specific points**	
Abstract: No need to include IRB details in the abstract.	IRB details removed.
In last line of the Results you have deleted "years" which needs to be put back for the sentence to make sense. Conclusion is poorly written with a phrase repeated twice in the same sentence. This is probably because a clean (i.e., no tracked) document wasn't ready through.	Amended.
Introduction: Literature has been updated, thank you	Noted with thanks.
Methods: You should include the vertical transmission guidelines and antenatal and postnatal care guidelines for context.<br><br>What did mothers' receive? Was there any post-exposure prophylaxis for infants? This needs to be taken into account in your models. If you summarise the Vertical Transmission of HIV Prevention and EID guidelines it would be	Summary of the guidelines given in the introduction.<br><br>This has been included in the study in the context of if the mother was on the first line ART regimen or the Second line ART regimen. This has also been factored in the analysis.

clear that there was NO BIRTH testing at the time of the study. This is an important point.	I have also added the information that all the infants were put on prophylaxis from birth (all were hospital deliveries.)<br>It has also been made clear that there was no birth testing at the time and that it was done between 4-6 weeks or at the earliest opportunity thereafter as per the guidelines.
The sample size calculation is still unclear; what proportion of vertical transmission was expected and why? The study was powered at 0.8 /80% to detect what? I still cannot determine whether the sample size is appropriate for the information presented.	Sample size calculation formulae included in the manuscript
Results: You need to report uncertainty ranges around the central tendency. This means and inter-quartile range around medians and SD or 95% CI around means.	I have removed this section and just reported the mothers ages.
Table 2: there is not enough information about the variables. E.g., age in completed years? To what age does the chi apply? The same applies to almost ALL these variables. Is there any reason why the actual numbers aren't included? Can't these data, not the chi values but the p-values, be included in Table1?	- Age is in completed years and this has been indicated<br>- Table one is a descriptive table that shows the demographic characteristics of the participants at recruitment and at the endpoint (This table was initially missing, and you recommended that I add it). -Table two shows a test of association at the end of the study. This was a longitudinal study and there was attrition of participants, hence the reason for the n=131.
Cox PH – should be spelled out	This has been modified
Is the old Table 5 on hypotheses deleted or not? Only the heading is deleted.	It is deleted.
Death and transfer out are competing events. i.e., you cannot become HIV infected if you die or if you transfer out and are not tested. You need to apply a statistical method to account for these competing events.	Cumulative incidence function was used to model for possible competing interests i.e. death and transfer outs.
Discussion: could be edited for length	Discussion edited. This has been shortened from 7 to about 4 pages
Limitations: Limitations not mentioned include:<br>1. Recruitment at 2 -6 weeks (there may be a good reason for this, but it has not been explained as the VTP guidelines in place at the time are not described in the Methods). We know that infants infected in utero can have rapid progression of HIV and die before 6 weeks old. Recruitment at 6 weeks will have missed infants with in utero HIV infection who died, under-estimating VT.<br>2. Breastfeeding – no data are presented. This should be noted as an important variable in	Limitations<br>1. The GOK policies indicate testing be done at 4-6 weeks and is hence not a study limitation per say... This has been alluded to in the manuscript. Birth testing has been piloted in some areas currently but has not been passed as a routine policy.<br>2. Data on breastfeeding rates have now been provided in the manuscript. However bivariable and multivariable analysis on the same was not done because there was no way to corroborate the feeding options for the infants from the mother's narrative, at the time

VT, especially post-natal VT. What are BF rates in the region?<br>3. Infant post-exposure prophylaxis – no data are presented. As above, this is an important variable in VT.	of the study. This has therefore been pointed out as a study weakness.<br>3. Infant prophylaxis has been alluded to in the manuscript. All the infants received prophylaxis from birth (all mothers delivered in the hospital and are given a baby pack that has prophylaxis for 6 weeks).
**REVIEWER 3**	**Changes effected by the PI**
In the results, you state that 'infant death was not a competing risk factor' - but this doesn't make sense to me, as death is an outcome (failure) in your analysis. Please confirm with a statistician that this analysis was adequately rigorous. And please make sure you define what the outcomes are in your methods section - in other papers in the region, any outcome other than a final HIV-negative test is considered *not* to be HIV-free survival - be very clear how you count 1. Death, 2. Loss-to-follow-up, and 3. HIV infection - ie. Are they all included as failures in your survival analysis	- Statistician consulted and Cumulative incidence function was used to model for possible competing interests i.e. death and transfer outs.<br>- The definition of the outcome of interest (HIV-free survival) has been defined in the introduction and in the methods. It was initially on the results section but advised by the second reviewer to move to the methods section.
The last sentence of your abstract introduction paragraph needs to move up to become the second sentence - the flow will be better then.	This has been amended as advised.
Your discussion is very long - consider reducing it by a few paragraphs and focusing on the 2-3 *key* messages you want the reader to remember. Then make sure your conclusion reiterates those main points	Discussion amended and shortened. This has been shortened from 7 to about 4 pages
Prepared by:<br>Dr. Elizabeth Kiilu<br>Tel: +254 721724580<br>Email: elisakiilu09@gmail.com<br>1st July 2023	
