# Supplementary material for: Prognostic factors influencing HIV-free survival among infants enrolled for HIV early infant diagnosis services in selected hospitals in Nairobi County, Kenya
Source: PLoS One. 2023 Oct 4;18(10):e0292427. doi: 10.1371/journal.pone.0292427 (PMC10550113; doi:10.1371/journal.pone.0292427)
Supplement: Attachment — Submitted filename: Response to Reviewers.docx [file pone.0292427.s003.docx]

| **Response to reviewers (23 JULY 2022)** | |
| --- | --- |
| **Reviewers Comments** | **Changes effected by the PI** |
| Please ensure that your manuscript meets PLOS ONE's style requirements, including those for file naming. | Document aligned and PLOS ONE style requirements |
| Please include additional information regarding the survey or questionnaire used in the study and ensure that you have provided sufficient details so that others could replicate the analyses. For instance, if you developed a questionnaire as part of this study and it is not under copyright more restrictive than CC-BY, please include a copy, in both the original language and English, as Supporting Information. | The questionnaire is attached as supporting documents |
| Furthermore, please provide additional information on the steps taken to test the validity of the question and details regarding how the questionnaire was developed. | Validity of data collection tools indicated in the methods |
| Finally,in your Methods section, please provide a justification for the sample size used in your study, including any relevant power calculations (if applicable). | Section inserted to show sample size and sampling technique, power of the study, and level of statistical significance highlighted. |
| Please state what role the funders took in the study. | Role stated i.e. "The funders had no role in study design, data collection, and analysis, decision to publish, or preparation of the manuscript. |
| Funding Information’ and ‘Financial Disclosure do not match.  When you resubmit, please ensure that you provide the correct grant numbers for the awards you received for your study in the ‘Funding Information’ section. | Role of funders explained. |
| Please include this amended Role of Funder statement in your cover letter; we will change the online submission form on your behalf. | Role of funders included in the cover letter |
| . We note that the grant information you provided in the ‘Funding Information’ and ‘Financial Disclosure’ sections do not match.  When you resubmit, please ensure that you provide the correct grant numbers for the awards you received for your study in the ‘Funding Information’ section. | The funding information section will be amended as I submit. |
| Funding information should not appear in the Acknowledgments section or other areas of your manuscript. We will only publish funding information present in the Funding Statement section of the online submission form.  Please remove any funding-related text from the manuscript and let us know how you would like to update your Funding Statement. Currently, your Funding Statement reads as follows: "YES National Research Fund- Government of Kenya: NRF/PhD/02/10  Jomo Kenyatta University of Science and Technology: JKU/ 4540(32)"  Please include your amended statements within your cover letter; we will change the online submission form on your behalf. | The funding statement was removed from the acknowledgment section. Information on the same included in the cover letter |
| In your Data Availability statement, you have not specified where the minimal data set underlying the results described in your manuscript can be found. PLOS defines a study's minimal data set as the underlying data used to reach the conclusions drawn in the manuscript and any additional data required to replicate the reported study findings in their entirety. All PLOS journals require that the minimal data set be made fully available. For more information about our data policy, please see <http://journals.plos.org/plosone/s/data-availability>.  Upon re-submitting your revised manuscript, please upload your study’s minimal underlying data set as either Supporting Information files or to a stable, public repository and include the relevant URLs, DOIs, or accession numbers within your revised cover letter  If there are ethical or legal restrictions to sharing your data publicly, please explain these restrictions in detail.  We will update your Data Availability statement to reflect the information you provide in your cover letter. | The minimal underlying data set had been provided as part of supporting files for reviewer access. The data set has been de-identified, however, the data set remains part of the Government of Kenya, Ministry of Health property, and permission to share it publicly needs to be sought from the director general using the following contacts:  Dr. Patrick Amoth  Ag. Director General for Health at Ministry of Health  Ministry of Health,  Afya House, Cathedral Road,  P.O. Box:30016–00100, Nairobi, Kenya.  Email: dghealth2019@gmail.com |
| . Your ethics statement should only appear in the Methods section of your manuscript. If your ethics statement is written in any section besides the Methods, please delete it from any other section. | The ethics statement only appeared in the ethics section |
| There were some typos in the document, so needs further proofreading. | Document proofread and typos corrected |
| I would prefer that when the authors are discussing other groups' work, then they say ...et al (instead of having a reference in the middle of a sentence). E.g. p19 "a finding corroborated by [2] whereby male partners who..." should be "a finding corroborated by Alusio et al [2] whereby male partners who..." | Section amended and citation put at the end of the paragraph |
| Please state over what time period the household income of Ksh. <6000 is for. Is it per annum? | Indicated i.e. monthly household income |
| The acknowledgments section needs to be re-written. E.g. Include study participants, health care providers, and funders. It is strange to acknowledge people that are authors of the study. It is written in a way that I would expect to be in a Ph.D. thesis, not a scientific manuscript. | Section edited and funding information removed from here. Co-authors also removed from this section |
| While revising your submission, please upload your figure files to the Preflight Analysis and Conversion Engine (PACE) digital diagnostic tool, <https://pacev2.apexcovantage.com/>. | Figures uploaded successfully to Preflight Analysis and Conversion Engine (PACE) |
| Prepared by:  Dr. Elizabeth Kiilu  Tel: 0721724550  Email: [elisakiilu09@gmail.com](mailto:elisakiilu09@gmail.com) | |
