# Supplementary material for: Prognostic factors influencing HIV-free survival among infants enrolled for HIV early infant diagnosis services in selected hospitals in Nairobi County, Kenya
Source: PLoS One. 2023 Oct 4;18(10):e0292427. doi: 10.1371/journal.pone.0292427 (PMC10550113; doi:10.1371/journal.pone.0292427)
Supplement: Attachment — Submitted filename: PONE-D-21-18949R1.docx [file pone.0292427.s004.docx]

**PONE-D-21-18949R1**

Manuscript Title: Prognostic factors influencing survival among infants enrolled for HIV early infant diagnosis services in selected hospitals in Nairobi County, Kenya

The authors present a longitudinal analysis of HIV infection in children who are HIV-exposed between 2-6 weeks and 12 months old in Nairobi, Kenya. The manuscript is not suitable for publication in its present form. I hope the suggestions below will strengthen any future submissions.

**General points**

1. The manuscript retains elements of a dissertation – selected headings, inclusion of textbook formulae, Table 5, the order of headings, amongst others…
2. This is a survival *analysis*, and the authors define the event as infant HIV-infection so the appropriate term is **HIV-free survival**. ‘Survival analysis’ implies alive/dead which is not the case and it is inappropriate to describe any variables as factors associated with *survival.* This needs to be amended in the Title and *throughout*.
3. The manuscript appears incomplete, particularly parts of the Methods and the Results sections and several Tables. There is *insufficient detail* to provide context to the study and lack of attention to detail in terms of presentation: headings, spelling out acronyms, mixing of tenses, lack of full sentences in the Methods, references referred to as a number in the Discussion, amongst others…
4. In the HIV field, there is a move towards using more patient-centred language and avoiding the terms mother-to-child-transmission (use ‘Vertical Transmission’) and HIV-infected/positive (use ‘living with HIV’).
5. Many of the references are outdated and the results have been super-ceded. The most recent reference is 2019. There is much more contemporary literature available.

The document requires complete revision. I suggest you read related peer-reviewed papers and model your manuscript on this presentation, in addition to abiding by PLOS One’s style requirements (it is not aligned).

**Specific points**

Title: As noted above the term ‘survival’ is misleading as it implies not dying. This is fundamental.

Abstract: This is not detailed enough to provide a comprehensive summary of the study. You should include the outcome variable and main exposure variables. No need to include IRB details in the abstract. The conclusion that factors are associated with ‘poor infant survival’ is false; these factors are associated with HIV infection.

Introduction: The references used to set context are out-dated and need to be updated. To my knowledge, Botswana has also achieved elimination of vertical transmission of HIV or is close. Not a single acronym is spelled out.

Methods: This section lacks important details. You should include the vertical transmission guidelines and antenatal and postnatal care guidelines for context. It is essential to include the early infant diagnosis testing protocols.

Some description of the study population is given but the connection between the description and why the study sites were selected is omitted.

Many sections are not written in full sentences.

Why were infants only recruited at 2-6 weeks? Was there any birth testing? If not, then why exclude the youngest group which may have been infected *in utero*?

The sample size calculation is unclear; what proportion of vertical transmission was expected and why? I cannot determine whether the sample size is appropriate for the information presented.

From where were the **PCR data** obtained? Lab results, self-report? Were all infants tested as per guidelines?

**Breastfeeding** is an important variable when considering vertical transmission of HIV after the first couple of weeks. I don’t see reference to breast-feeding.

‘Study Validity’ section: this does not describe validation of the questionnaire but a pilot study. A validation study is completely different and requires comparison against a gold standard.

The formulae on pages 9, 10 and 11 are unnecessary.

You need to report uncertainty ranges around the central tendency.

Ethics: it is not clear that IRB approval was actually granted.

Limitations: This is an important section and belongs AFTER the Discussion.

It is suggested here that there may be **HIV exposure misclassification**. In other words, it could not be confirmed that all enrolled infants were actually HIV-exposed as per the inclusion criteria. This is a major limitation and calls into question the results. This should have been dealt with in the study design in that only infants with a confirmed HIV-exposure status should have been enrolled.

Results: Present the descriptive data first and have a table of baseline characteristics at enrolment.

How many were screened, over what period etc.

Present the actual numbers and proportions.

Death and transfer out are **competing events.** i.e., you cannot become HIV infected if you die or if you transfer out and are not tested. You need to apply a statistical method to account for these competing events.

Table 1: present actual numbers and proportions with the p-value. The Chi statistic and degree of freedom are not required in the table.

Tables 2 and 3 seem to be missing most of their data.

Table 5 doesn’t belong in the results section of a manuscript

The 95% CI are very wide.

Discussion: It is difficult to comment as the results are not clearly presented.

Results are referenced for the first time in the discussion.

It is not usual practice to refer to a reference number in the text. It is preferable (required) to integrate the relevant content from the reference in words in the text.

References: need updating

The internet references and reports are incomplete.
