# Supplementary material for: Prognostic factors influencing HIV-free survival among infants enrolled for HIV early infant diagnosis services in selected hospitals in Nairobi County, Kenya
Source: PLoS One. 2023 Oct 4;18(10):e0292427. doi: 10.1371/journal.pone.0292427 (PMC10550113; doi:10.1371/journal.pone.0292427)
Supplement: Attachment — Submitted filename: PLosONE_KE_EID_Review_oct2022.docx [file pone.0292427.s005.docx]

**Review:** Prognostic factors influencing survival among infants enrolled for HIV early infant diagnosis services in selected hospitals in Nairobi County, Kenya

**Note: I have a lot of feedback in this review – you’ve got potentially a great paper, but it needs a lot of work – read through my comments a few times and consult your advisor(s) before tackling the changes one at a time.*

Overall –

- “HIV-positive” and “HIV-infected” are no longer politically correct terminology; suggest using “women living with HIV (WLHA) throughout”. Also suggest using “HIV-exposed infants (HEI)” as they are the subject of a growing field of research – this will make your publication easier to find for those doing literature reviews
- The sub-headings in the paper read like a thesis or a dissertation – specifically, the methods section has many more sub-headings than needed for a manuscript. I suggest looking at similar papers in the literature (especially within this journal), and see what subheadings are consistently used. Style your paper the same way; it’s okay if not everything fits in.

Title –

- Why ‘prognostic’? Suggest removing the word throughout the paper and just say ‘factors’. Your title indicates survival is the end-point, but your methods indicate HIV-positive status as the endpoint. Needs to be clear if it’s one or the other or either.

Abstract –

- Intro - ‘MTCT rate’ needs to be presented with an end time (ie. At 6 weeks of age; 12 months of age; after weaning) – also, technically a rate is measured in person-years (as you’ve done in your results). Suggest using ‘percentage’ or ‘proportion’ in this context
- Methods – Number of facilities? Public and/or private? Sample selected from how many HIV-infected infants (as denominator for proportion chosen). Why one year? Were they all weaned by then, or is the in the context of ongoing exposure into the second year of life? What data was collected? Maternal only (as implied in the conclusion of the abstract? Or infant as well, ie. Birthweight, feeding method, weaning age, etc. Ethical approval numbers not needed in the abstract
- Results – failure event definition belongs in your methods (but isn’t needed in the abstract if space is a challenge). What is “poor infant survival”? Is that death and HIV-infection? Just death? Needs to be very clear. At the moment it reads as though you’re using death and HIV-infection interchangeably and conflating the two into ‘poor infant survival’
- Conclusion – why define recent HIV diagnosis in relation to the ‘study onset’. Rather define it as it relates to her own condition and health: ie. ‘less than two years before current pregnancy’.

Introduction

- Always spell out the first use of acronyms in the main manuscript: HIV, EID, PMTCT – even if spelled out already in the abstract
- This is very short and doesn’t provide a solid introduction to the topic or the other research in this area. The sentence “there is limited data demonstrating…” in the first paragraph is inaccurate. There’s A LOT of research in both MTCT and infant death; you need to use that to your benefit – understand what’s out there and what your study brings to the table that is 1. New or additional in the literature, or 2. Confirms what others have found in other countries holds true in Kenya as well
- This first paragraph is extremely confusing – there’s actually more literature on MTCT than on HIV exposed/infected infant survival. This wording doesn’t help clarify for the reader immediately which direction you’re going in -the implication is HIV infection, but your paper title is “survival” – if your argument later in the paper is to do a survival analysis using HIV infection as your end point, that’s different that saying ‘survival’ and meaning HIV-negative. The ‘survival’ in ‘survival analysis’ by definition is the opposite of your end point. But if your end point is HIV-infection, then your ‘survival’ is ‘HIV-free’; you can’t use ‘survival’ in the title or narrative as anything other than being alive; you must use ‘HIV-free’ or ‘HIV-negative’, or the reader is left confused
- Your references are limited to East Africa research – suggest you broaden your look to southern Africa as well. A lot of PMTCT research (including national level evaluations) have been reported from Malawi, Zimbabwe and South Africa – many of which have similar analyses as yours. Understanding their findings and how yours do or don’t differ will be important to the interpretation of your results section in your discussion
- Consider your key findings and think about how the reader needs to be prepared to accept those – ie. Disclosure between partners is really important – there’s a paper out of Malawi’s national PMTCT evaluation that documents the same, and many other papers showing that male involvement from early in ANC and couples testing is also critical to maternal ART adherence… which of course is what infant infection hinges on – bring these ideas together, along with other key findings.
- The paragraph that begins with “Globally, vertical transmission” should be the first paragraph in your paper – it sets the stage for everything else – always start your introduction with the global setting and then narrow down to national and if relevant, sub-national levels. That first sentence also needs to be broken into two sentences..
  - Suggestion: This diagram (sourced online) is used widely to describe what the flow of your introduction should look like:

- What is currently the 1^st^ and 2^nd^ paragraph needs rethinking – move it further down to after you’ve provided the global and Kenya contexts for MTCT / HIV-free survival
- Be careful you don’t put methods in your intro – ie. “survivorship in this study was defined as…”
- The paragraph starting “In Kenya” isn’t connected to anything – what’s the purpose of this? If you think it’s a necessary piece of ‘telling your story’, it needs to fit in with the flow and purpose of the paper

Methods

- Sub-headings – should be same size font as the narrative, not bolded, and in italics.
- Main headings (ie. Intro, methods) – should be same size font as narrative
- If you need a third level heading, make it the same font as the narrative and underline it (no bold or italics)
- Female prevalence compared to male prevalence isn’t relevant in your methods section – probably doesn’t need to be in background either – suggest removing
- The details about prevalence in catchment areas could be part of your background section where you talk about Kenya and what’s specifically known/unknown about HIV and MTCT in your study area. Don’t put site names in your intro; keep those in your methods
- “infants born of HIV-positive mothers” should be “HIV-exposed infants”
- Sampling – rename subsection to “Study population”
- “You need to explain why you chose to use the EID clinic as entry point for the study – why not ANC? Also make sure you include this as a limitation in your discussion section. Your sample excludes infants not identified as HIV-exposed (whether or not their mothers were on ART), and infants who may have died prior to enrolment.
- Define CCC
- Once the participants were identified from the register, how did you identify them in real life to enroll them? A little more detail is needed here
- Exclusion criteria – why exclude those who transferred in within the last year?
- Consider putting your inclusion criteria into a single sentence, and your exclusion criteria into a single sentence – think about how to be more concise – ie. “HIV-exposed infants age 2-6 weeks whose mothers were registered in the study facility CCCs and agreed to participate in the study” – three sentences are not needed.
- Study variables – you don’t need a section listing these out – combine with the data collection tools section and be more clear about what was collected from routine (MOH) data, what was collected from your questionnaire, and what labs were collected (or include with routine MOH data if testing was not done separately for the study)
- Cohort recruitment and follow-up – move up to come after the sampling section to improve flow
- Study validity – you describe what you did and why, but not “how” – did you do any statistical testing for validity? If not, consider removing this section entirely
- Data analysis –
  - This section needs to describe everything and only what you did – no excess language or definitions of stats techniques. You’re missing a description of what you did that aligns with the multiple tables produced in your results:
  - Your first paragraph is almost all that’s needed: Consider using “conducted” instead of “done” in the second use of “done”; Revise to remove brackets – ie. Remove (mean and median), and reorganize the wording in the K-M sentence (‘Kaplan-Meier survival analysis provided…’)
  - Remove the definition of Cox’s regression and all the formulas – these are standard statistical techniques and can be looked up online by anyone who doesn’t know them
  - ‘Modeling’ section – this can all be removed. Add AIC as one of your analysis techniques in the first (only) paragraph if wanted.
  - **Note:** Your methods data analysis description don’t match what you show in your results –
    - Why did you separate your multivariable analysis into two separate groupings after the log-rank test? Why not a single model?
      - And related, why were you limiting yourself to 2 factors in the final model?
    - That should be revised into a one comprehensive model, or alternately, describe what you did and why in this section… you’ve got this already, but you put these methods in your results section – move the first half of the paragraph that begins “all significant variables at p<0.01…” to your methods section. And then fill in the gaps in my understanding based on the questions immediately above.
  - Why “…to determine the best model using only two prognostic factors”? Why not all variables produced by the model?
- Study strengths and limitations (remove delimitations) –
  - This should be 1-2 paragraphs in your discussion section and not part of your methods
  - ‘Wasting’ is a chronic condition – not subject to ‘rapid fluctuations’. I suggest you stick to just saying ‘nutrition parameters’
  - If you have a study limitation, you need to think about how it might have affected your results – ie. You recruited at EID entry point so you have almost certainly underestimated HIV transmission and mortality because you didn’t include stillbirths and neonates – this also means you may have overestimated HIV-free survival (and proportion HIV-negative), which would explain why your results are higher than other countries…. These are the kinds of explanations to include in your limitations section.
  - A strength you need to include is that you used survival analysis as appropriate for a cohort study, instead of logistic regression as is often done with other cohort studies in this field
  - Sample size could influence your results up OR down – you only describe it one way. I think you can reframe this as the small sample size gave you wide confidence intervals (5.5-16.3) which isn’t as useful for health policy guidance as narrow CI’s.
- Ethical considerations – the sentence about women’s age should be moved up to your study population section
  - Also change your language to be more clear – not ‘sought approval’, but ‘received approval’. There’s a big difference. This section really only needs the listing of who provided ethical approvals, and the approval numbers where relevant

Results:

- In your results, you ONLY present results – no methods, no opinions, no interpretation
- This wording: “the study applied right-censoring”, and “K-M survival estimate curves were used…”, and “K-M failure estimate curves were used…” , and Mantel log-rank test was used…” all belongs in your methods section
- You’re missing a “Table 1” which provides the basic demographics for mother and infant (age, marital status, education, viral load at enrolment, birthweight, age at weaning, etc)- you need to describe the study population before you jump into the outcomes of the study
- When you describe a table in your narrative, you need to actually describe the content of the table – not just repeating the table title. Pick the 2-3 most important things from each table and describe them in your results narrative. When you describe, don’t repeat all the numbers in the table, but instead writing statements such as “nearly half the infants born to women under age 25 were lost to follow-up, compared to one quarter of infants born to women over age 35” (numbers I made up).
- Table 1 (should become 2) –
  - You need to ‘tab’ your unbolded row titles so they’re indented from the sub-headings within the table
  - Remove “Mantel log-rank tests for” from each sub-heading as the table title says this already
  - What is “timelines into EID enrolment at 6 weeks”? Is this about whether they were enrolled at a later age? If so, what cut-off did you use to determine late enrolment? 6 weeks? 12 weeks? Suggest revising this so it’s clear to the reader
- Table 2 –
  - Be careful with your formatting – keep a space between numbers and brackets for consistency – also indent the values from the variable names such as described above for Table 1(2). I
  - t’s not clear to me why you have two separate Cox regressions – is Table 2 from the enrolment data and Table 3 from 12 mos? If so, the table title is really misleading – I’m guessing from the variables included.
- Table 3 –
  - By now the reader is really confused as to what you’re doing – this process of presenting your results piecemeal wasn’t described in your methods
- Table 4 –
  - This is the ‘nyama’ of your multivariable regression. I suggest the other two regression tables aren’t needed – once you’ve described the process in your methods section (as advised above), then this is really the table that you want to talk about
  - This looks like an unusual way of presenting multivariable analysis results – I suggest reviewing other published papers and identifying a ‘standard’ presentation of Cox regression results
- Research hypothesis and test result findings – remove this section completely (and note – you don’t describe Fishers and Poisson in your methods, but Mantel log-rank and Cox – details matter!)

Discussion/Conclusion:

- More is not better: You need to decide what your absolutely critical takeaway message is for you paper – you’ve currently got multiple messages in here and nothing comes out clearly as the main point
- This should be about 2 pages long – most of what you have in here now actually belongs in your introduction section, prime examples being the paragraphs which start:
  - “Partner involvement…”
  - “A retrospective study in Tamil…”
  - “In rural Kenya…”
- Don’t repeat study definitions in your discussion section
- The second half of paragraph 1 is written nicely – you’ve clearly reframed your findings and compared them to regional findings – you don’t need to add all the CI’s though – you can just say “92% in Rwanda and 73% in Cameroon” for example
- More is not better: Keep language tight and straightforward - ie – on p20, “Further, add to say that detection of maternal HIV status” becomes “Detection of maternal HIV status”.
