# Supplementary material for: Prognostic factors influencing HIV-free survival among infants enrolled for HIV early infant diagnosis services in selected hospitals in Nairobi County, Kenya
Source: PLoS One. 2023 Oct 4;18(10):e0292427. doi: 10.1371/journal.pone.0292427 (PMC10550113; doi:10.1371/journal.pone.0292427)
Supplement: Attachment — Submitted filename: Response to Reviewers.docx [file pone.0292427.s007.docx]

| **Response to reviewers 24/06/2023** | |
| --- | --- |
| **Reviewers Comments** | **Changes effected by the PI** |
| **REVIEWER 2** |  |
| **General comments** | |
| It is suggested that the discussion section be shortened | This has been shortened from 7 to about 4 pages |
| No clean (i.e., not tracked) copy was included in the submission. This has made it difficult to read. | A clean version will be provided. |
| Font size for headings is still inconsistent | All heading font size made consistent. |
| In the HIV field, there is a move towards using more patient-centered language and avoiding the term mother-to-child-transmission (use ‘Vertical Transmission’). This has not been addressed in the Abstract or Manuscript. | In Kenya, the program is still called Prevention of Mother to Child Transmission (PMTCT), I will retain this while making reference to the program/clinic. I have however changed from PMTCT to vertical transmission of HIV while referring to the vertical transmission in the discussions.  This is mainly because you cannot call it a prevention of vertical transmission of HIV clinic, it's still called a PMTCT clinic/Program. |
| Breastfeeding is an important variable when considering vertical transmission of HIV after the first couple of weeks. I don’t see reference to breast-feeding | Reference on breastfeeding has been included. |
| Infant post-exposure prophylaxis is also an important variable in vertical HIV transmission and is not mentioned, not even the guidelines | This has now been reported |
| **Specific points** | |
| Abstract: No need to include IRB details in the abstract. | IRB details removed. |
| In last line of the Results you have deleted “years” which needs to be put back for the sentence to make sense.  Conclusion is poorly written with a phrase repeated twice in the same sentence. This is probably because a clean (i.e., no tracked) document wasn’t ready through. | Amended. |
| Introduction: Literature has been updated, thank you | Noted with thanks. |
| Methods: You should include the vertical transmission guidelines and antenatal and postnatal care guidelines for context.  What did mothers’ receive? Was there any post-exposure prophylaxis for infants? This needs to be taken into account in your models. If you summarise the Vertical Transmission of HIV Prevention and EID guidelines it would be clear that there was NO BIRTH testing at the time of the study. This is an important point. | Summary of the guidelines given in the introduction.  This has been included in the study in the context of if the mother was on the first line ART regimen or the Second line ART regimen. This has also been factored in the analysis.  I have also added the information that all the infants were put on prophylaxis from birth (all were hospital deliveries.)  It has also been made clear that there was no birth testing at the time and that it was done between 4-6 weeks or at the earliest opportunity thereafter as per the guidelines. |
| The sample size calculation is still unclear; what proportion of vertical transmission was expected and why? The study was powered at 0.8 /80% to detect what? I still cannot determine whether the sample size is appropriate for the information presented. | Sample size calculation formulae included in the manuscript |
| Results: You need to report uncertainty ranges around the central tendency. This means and inter-quartile range around medians and SD or 95% CI around means. | I have removed this section and just reported the mothers ages. |
| Table 2: there is not enough information about the variables. E.g., age in completed years? To what age does the chi apply? The same applies to almost ALL these variables. Is there any reason why the actual numbers aren’t included? Can’t these data, not the chi values but the p-values, be included in Table1? | - Age is in completed years and this has been indicated  - Table one is a descriptive table that shows the demographic characteristics of the participants at recruitment and at the endpoint (This table was initially missing, and you recommended that I add it). -Table two shows a test of association at the end of the study. This was a longitudinal study and there was attrition of participants, hence the reason for the n=131. |
| Cox PH – should be spelled out | This has been modified |
| Is the old Table 5 on hypotheses deleted or not? Only the heading is deleted. | It is deleted. |
| Death and transfer out are competing events. i.e., you cannot become HIV infected if you die or if you transfer out and are not tested. You need to apply a statistical method to account for these competing events. | Cumulative incidence function was used to model for possible competing interests i.e. death and transfer outs. |
| Discussion: could be edited for length | Discussion edited. This has been shortened from 7 to about 4 pages |
| Limitations: Limitations not mentioned include: 1. Recruitment at 2 -6 weeks (there may be a good reason for this, but it has not been explained as the VTP guidelines in place at the time are not described in the Methods). We know that infants infected in utero can have rapid progression of HIV and die before 6 weeks old. Recruitment at 6 weeks will have missed infants with in utero HIV infection who died, under-estimating VT. 2. Breastfeeding – no data are presented. This should be noted as an important variable in VT, especially post-natal VT. What are BF rates in the region? 3. Infant post-exposure prophylaxis – no data are presented. As above, this is an important variable in VT. | Limitations   1. The GOK policies indicate testing be done at 4-6 weeks and is hence not a study limitation per say... This has been alluded to in the manuscript. Birth testing has been piloted in some areas currently but has not been passed as a routine policy. 2. Data on breastfeeding rates have now been provided in the manuscript. However bivariable and multivariable analysis on the same was not done because there was no way to corroborate the feeding options for the infants from the mother's narrative, at the time of the study. This has therefore been pointed out as a study weakness. 3. Infant prophylaxis has been alluded to in the manuscript. All the infants received prophylaxis from birth (all mothers delivered in the hospital and are given a baby pack that has prophylaxis for 6 weeks). |
| **REVIEWER 3** | **Changes effected by the PI** |
| In the results, you state that 'infant death was not a competing risk factor' - but this doesn't make sense to me, as death is an outcome (failure) in your analysis. Please confirm with a statistician that this analysis was adequately rigorous. And please make sure you define what the outcomes are in your methods section - in other papers in the region, any outcome other than a final HIV-negative test is considered *not* to be HIV-free survival - be very clear how you count 1. Death, 2. Loss-to-follow-up, and 3. HIV infection - ie. Are they all included as failures in your survival analysis | - Statistician consulted and Cumulative incidence function was used to model for possible competing interests i.e. death and transfer outs. - The definition of the outcome of interest (HIV-free survival) has been defined in the introduction and in the methods. It was initially on the results section but advised by the second reviewer to move to the methods section. |
| The last sentence of your abstract introduction paragraph needs to move up to become the second sentence - the flow will be better then. | This has been amended as advised. |
| Your discussion is very long - consider reducing it by a few paragraphs and focusing on the 2-3 *key* messages you want the reader to remember. Then make sure your conclusion reiterates those main points | Discussion amended and shortened. This has been shortened from 7 to about 4 pages |
| Prepared by:  Dr. Elizabeth Kiilu  Tel: +254 721724580  Email: [elisakiilu09@gmail.com](mailto:elisakiilu09@gmail.com)  1^st^ July 2023 | |
