# Supplementary material for: Prognostic factors influencing HIV-free survival among infants enrolled for HIV early infant diagnosis services in selected hospitals in Nairobi County, Kenya
Source: PLoS One. 2023 Oct 4;18(10):e0292427. doi: 10.1371/journal.pone.0292427 (PMC10550113; doi:10.1371/journal.pone.0292427)
Supplement: Attachment — Submitted filename: Response to Reviewers.docx [file pone.0292427.s009.docx]

| **Response to reviewers 24/06/2023** | |
| --- | --- |
| **Reviewers Comments** | **Changes effected by the PI** |
| **REVIEWER** |  |
| **Introduction** | |
| Remove and re-edit the statement from in 2017 to Oman | Removed and reworded |
| Italicize the guidelines | This has been done |
| Country not county | It's actually county. The country has 47 counties |
| What period? Revise this | This has been revised |
| Proposed by whom? | Edited |
| Attach paragraph to previous sentences | Attached |
| Survivorship needs to go into the methods | Moved to the methods |
|  |  |
| **Methods** | |
| Kibra Spelling correct | corrected |
| Include generalizability as a weakness | Included |
| Correct wording for sample size | Corrected |
| Sample size not translating well in the pdf | Sample size will be anchored into the pdf. The sample size calculation has been rechecked by the statistician and is ok. |
| This group is possibly also at higher risk of transmitting HIV () The excluded group | The wording was not put well, hence it has been reworded to reflect that the records are available, but it takes time to transfer then to new their new centers |
|  |  |
| **Results** |  |
| First statement belongs to the methods | Has been moved to methods |
| Define LTFU in first instance | LTFU has been defined in the methods |
| Table 1 centered | Done |
| Remove frequencies and write % | This has not been done as was recommended to leave it with n (%) in previous reviews |
| Check grammar page 12 and be concise | Corrected |
| Failure event definition to be removed in results | Corrected |
| Corrections on page 13 | Amended |
| Table 2 | Amended |
| Define AHR and HR the first time they appear | This has been defined in the methods. However this may not be necessary to have as this would mean you may also need to define other statistical terms. All the same the definition has been given. |
| Table 3 why have you separated demographic characteristics from clinical characteristics....run as separate models... | They were only separated as they could not fit on the same table but analysis was not run separately. Table has now been merged to avoid that confusion. |
| Table 5 looks like repeated analysis of table 4 | Following discussions with the statistician it was decided that there was no need to repeat these variables as they had already been shown in table 4. |
| **Discussion** |  |
| Paragraph 1should be concise and a summary of your key findings, subsequent paragraphs you can start comparing findings in other countries. | Amended |
| It's good to compare findings of other countries ...give narrative for your finding | Done |
| Be creative with your wording | Amended |
| Double check reference 20 | Amended appropriately |
| Limitations | Have been updated appropriately |
| **Conclusion** |  |
| Remove more so | Amended |
| General concern from author: Some parts of the comments have overlapping texts and hence were not legible, and some were not visible to the end. I have tried my best to interpret those comments that had this challenge. | |
| Prepared by:  Dr. Elizabeth Kiilu  Tel: +254 721724580  Email: [elisakiilu09@gmail.com](mailto:elisakiilu09@gmail.com)  21^st^ August 2023 | |
